# The Role of Neuroinflammation and Network Anomalies in Drug-Resistant Epilepsy

**DOI:** 10.1007/s12264-025-01348-w

**Published:** 2025-02-24

**Authors:** Jianwei Shi, Jing Xie, Zesheng Li, Xiaosong He, Penghu Wei, Josemir W Sander, Guoguang Zhao

**Affiliations:** 1 Department of Neurosurgery, Xuanwu Hospital, Capital Medical University, Beijing, 100053 China; 2 China International Neuroscience Institute, Beijing, 100053 China; 3 Deanery of Biomedical Sciences, Edinburgh Medical School, College of Medicine and Veterinary Medicine, University of Edinburgh, Edinburgh, EH8 9AG UK; 4 Department of Psychology, University of Science and Technology of China, Hefei, 230022 China; 5 Department of Clinical and Experimental Epilepsy, UCL Queen Square Institute of Neurology, London, WC1N 3BG UK; 6 Chalfont Centre for Epilepsy, Chalfont St Peter, Buckinghamshire, SL9 0RJ UK; 7 Neurology Department, West China Hospital of Sichuan University, Chengdu, 61004 China

**Keywords:** Neuroglia, Neuro-immune interaction, Brain network, Chronicity, Epilepsy

## Abstract

Epilepsy affects over 50 million people worldwide. Drug-resistant epilepsy (DRE) accounts for up to a third of these cases, and neuro-inflammation is thought to play a role in such cases. Despite being a long-debated issue in the field of DRE, the mechanisms underlying neuroinflammation have yet to be fully elucidated. The pro-inflammatory microenvironment within the brain tissue of people with DRE has been probed using single-cell multimodal transcriptomics. Evidence suggests that inflammatory cells and pro-inflammatory cytokines in the nervous system can lead to extensive biochemical changes, such as connexin hemichannel excitability and disruption of neurotransmitter homeostasis. The presence of inflammation may give rise to neuronal network abnormalities that suppress endogenous antiepileptic systems. We focus on the role of neuroinflammation and brain network anomalies in DRE from multiple perspectives to identify critical points for clinical application. We hope to provide an insightful overview to advance the quest for better DRE treatments.

## Introduction

Up to one-third of people with epilepsy have drug-resistant epilepsy (DRE), characterised by continuous seizures and often progressive cognitive decline [1, 2]. New anti-seizure medications (ASMs) launched in the last three decades have failed to reduce the prevalence of DRE [1]. Neuroinflammation, activated by various immune components such as reactivated glia, cytokines, and chemokines, involves numerous neural regulatory processes. This includes neurogenesis, cell proliferation, differentiation, and migration, and it may also play a role in DRE [3–5]. A growing body of evidence suggests that dysfunction of microglia and astrocytes, located in epileptic foci, notably in the hippocampus, plays a pivotal role in neuroinflammation and hyperexcitable neuronal networks in DRE [6–8]. As brain-resident immune cells, microglia primarily mediate inflammation and neuronal survival [9]. Aberrant synaptic pruning by activated microglia can lead to a synaptic excitatory/inhibitory imbalance [10]. After brain insults, reactive astrocytes release pro-inflammatory cytokines, recruit inflammatory cells, and cause abnormal neural connectivity through extensive biochemical changes such as connexin (Cx) hemichannel excitability and disruption of neurotransmitter homeostasis [11, 12]. Synaptic remodelling driven by reactive or inflammatory astrocytes and the disruption of GABAergic neural networks may play a key role in the reorganisation of neural circuits in epilepsy. This mechanism merits further investigation [13]. Overall, neuroinflammation may be essential for seizures and their resistance to pharmacological interventions.

More than 100 billion distinct neurons interact with inconceivable complexity and precision. Within the neuronal network, specific signalling molecules can trigger abnormal growth of neurons, such as axonal sprouting, synaptic reorganisation, and neurogenesis [14]. These modifications may result in network anomalies, complicating the ability of classical ASMs to target excitatory and inhibitory mechanisms effectively, which could contribute to drug-resistance development. The structural and functional dysconnectivity in brain networks caused by inflammation have been discussed [15, 16]. In the evolving field of network neuroscience, topological brain connectivity has emerged as a pioneering approach to managing DRE [17]. Epilepsy surgery is a treatment option for some people with DRE. Well-screened candidates for epilepsy surgery have seizure-free rates of 50–80% [18]. Continuous improvements in presurgical evaluations are essential to increase access to curative surgery. Symptomatology and neuropsychology are important methods in the presurgical evaluation of epilepsy but have some degree of subjectivity and limited accuracy. Non-invasive presurgical assessment, such as magnetic resonance imaging (MRI), scalp electroencephalography (EEG), magnetoencephalography (MEG), and positron emission tomography (PET), have significantly advanced the presurgical workup by providing increasingly precise proxies for identifying epileptogenic zones. Conversely, intracranial recordings (neural probes) can help with electrocorticography and stereo-electroencephalography (SEEG). Beyond epileptogenic zone identification, the accurate acquisition of neural signals, decoding system codes, and the bidirectional interaction of brain-computer interface (BCIs) might be used to predict seizures' type, onset, duration, and termination [19–22]. Non-invasive modulation techniques such as transcranial direct current stimulation (tDCS) and transcranial magnetic stimulation (TMS), together with chronic invasive neuromodulation, may also exert modulatory effects at different levels of neurophysiology (molecular, cellular, and network). Removal of the epileptogenic zone remains a cornerstone in managing DRE; but neuromodulatory strategies provide less invasive alternatives, especially for those non-suitable candidates. A better understanding of the networks will improve neuro-technologies and devise closed-loop treatment strategies for DRE.

Here, we discuss the impact of neuroinflammation on elucidating the pathomechanisms underlying DRE, particularly emphasising the significant role of glial cells. We explore the correlation between neuroinflammation and neuronal network dysfunction in DRE. The advances in network neuroscience are illustrated by a range of ingeniously designed neuronal devices that may mitigate the harmful effects of DRE. We advance some thoughts to explain the intractable characteristics of DRE.

## Neuro-immune Crosstalk in DRE: An Elusive Companion

**Fig. 1:**
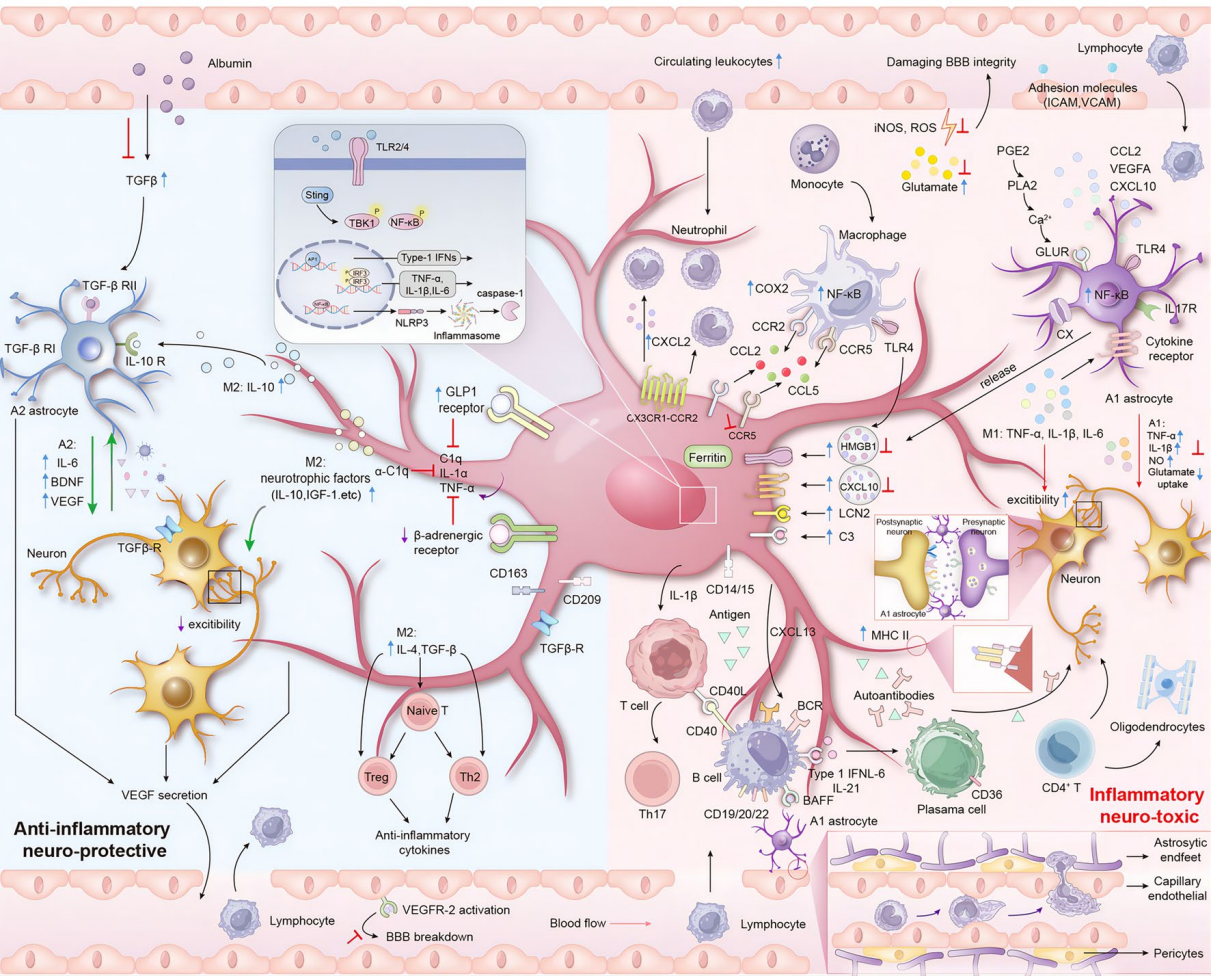
Characteristics of the neuro-immune microenvironment in DRE. The immune landscape in DRE is centred on microglial activation. On the right, M1 microglia and A1 astrocytes amplify neuroinflammation, contributing to neurotoxicity. On the left, M2 microglia and A2 astrocytes exert neuroprotective functions, secreting anti-inflammatory mediators like IL-10 and TGF-β, which modulate astrocytic activity [45, 255]. A2 astrocytes release neurotrophic factors such as BDNF, CLCF1, and IL-6 to mend damaged synapses and promote neuronal survival [45, 255]. Neuroprotective microglia facilitate Th2 and Treg cell differentiation *via* IL-4 and TGF-β cytokines [255]. VEGF enhances brain perfusion, partly by fostering angiogenesis [256]. Notably, the roles of neuroprotection and neurotoxicity are context-dependent: while IL-6 and TGF-β can serve as neuroprotective agents, they also exacerbate epileptic activity. Pathological VEGF levels compromise the BBB and trigger aberrant vascularisation [257]. M1 microglia and A1 astrocytes orchestrate a complex neuroinflammatory landscape by releasing cytokines like TNFα, IL1β, and IL6, which activate glial cells and promote leukocyte infiltration *via* endothelial adhesion molecules [102]. This milieu is further complicated by HMGB1, which exacerbates hyperexcitability and epilepsy through the TLR4 and IL-1β/NF-κB pathways [102–105]. Concurrently, upregulation of COX-2 triggers excessive PGE2 production, inducing drug resistance and secondary neurotoxicity [102]. Pericytes contribute by secreting chemokines (CCL2, CXCL1, CXCL8, and CXCL10) that recruit leukocytes, facilitated by endothelial ICAM-1 and VCAM-1 [28, 224, 257]. Monocyte and macrophage recruitment are mediated by M1 microglia through CXCR1-CCR2 and CCR2/CCR5 receptors, respectively. CD4+ T cells directly interact with oligodendrocytes [31, 32, 86]. This intricate network culminates in an imbalance between glutamatergic and GABAergic signalling, lowering the seizure threshold and perpetuating neuroinflammation [25, 207]. In summary, categorising chronic inflammation in DRE as a mere phenotypic shift in astroglia and microglia from anti-inflammatory, cytoprotective roles to pro-inflammatory, cytotoxic functions oversimplifies a potentially more complex landscape. (Red T-bars, the candidate inhibitory mechanisms of neuroinflammation; red arrows, neurotoxic effects; green arrows, neuroprotective effects. Abbreviations: DRE, Drug-Resistant Epilepsy; IL, Interleukin; TGF-β, Transforming Growth Factor Beta; BDNF, Brain-Derived Neurotrophic Factor; CLCF, Cardiotrophin-Like Cytokine Factor; VEGF, Vascular Endothelial Growth Factor; BBB, Blood-Brain Barrier; CX, connexin; iNOS, Inducible Nitric Oxide Synthase; ROS, Reactive Oxygen Species; PGE2, Prostaglandin E2; MHC, Major Histocompatibility Complex; HMGB1, High Mobility Group Box 1; TLR, Toll-Like Receptor; COX, Cyclooxygenase; CCL, C-C Motif Chemokine Ligand; CXCL, C-X-C Motif Chemokine Ligand; ICAM, Intercellular Adhesion Molecule; VCAM, Vascular Cell Adhesion Molecule; CXCR, C-X-C Chemokine Receptor; CCR, C-C Chemokine Receptor; GABA, Gamma-Aminobutyric Acid).

The intersection of immune responses and epileptogenesis has obtained attention over the years [23]. Cerebral tissue obtained through resection surgery in people with DRE has shown inflammatory responses, including altered complement pathway activity and blood-brain barrier (BBB) leakage [24, 25]. The immune microenvironment of DRE is complicated as it involves a dynamic interplay of inflammatory mediators, endothelial components, peripheral immune responses, neurons, and CNS-resident glial cells. Understanding the neuro-immune crosstalk is crucial in unravelling the mechanisms underlying drug resistance and devising effective therapeutic interventions (Fig. 1).

### Peripheral Innate Immune Cell Extravasation in the Brain

#### BBB Leakage

Seizure activity triggers transformative changes in the BBB structure and activates inflammatory pathways, oxidative stress, and efflux proteins such as P-glycoprotein (Pgp) [26]. As crucial constituents of the BBB, pericytes have gained prominence for their multifaceted roles in neuroinflammation [27, 28]. They secrete interleukin-6 (IL-6) to skew microglia toward a pro-inflammatory state and upregulate endothelial adhesion molecules (intercellular adhesion molecule 1 and vascular cell adhesion molecule 1) to facilitate leukocyte infiltration into the CNS [27, 28]. Concurrently, pericytes release reactive oxygen/nitrogen species, nitric oxide, and prostaglandin E2 (PGE2), causing vasodilation and compromising the BBB [27, 28]. PGE2 induces secondary neurotoxicity and elevates Pgp levels, imparting drug resistance *via* the EP1 receptor. Through the EP2 receptor, PGE2 signalling exacerbates and sustains neuroinflammatory cascades by enhancing pro-inflammatory mediators. Previous studies have demonstrated that COX-2 activation contributes to pharmacoresistance by inducing the transcription of Pgp (also called multidrug resistance gene) in brain vessels [29], and TLR4 receptors also play a role in activating MDR gene transcription [30], highlighting the importance of the BBB and neuroinflammation in drug resistance.

#### Monocytes and Neutrophils

Opening of the BBB, which can occur due to epileptic seizures, enables the invasion of circulating monocytes into brain tissue, thereby exacerbating neuro-inflammation. Preclinical models of acute brain injury, including status epilepticus (SE), also suggest that BBB disruption may precede the onset of seizures. It has been ascertained that suppressing CCR2+ monocyte recruitment into the brain can lessen BBB degradation and neuronal injury, suggesting potential therapeutic implications [31]. Following SE, monocyte infiltration significantly promotes microgliosis [32]. A clear differentiation between macrophages derived from infiltrated monocytes and activated microglia within the CNS parenchyma is essential for understanding these cells' diverse roles [33]. In a pilocarpine-induced SE model, flow cytometric analyses showed that the early-infiltrating leukocytes in the brain are neutrophils (CD11b+CD45high, Ly6G+Ly6C+), thereby intensifying acute neuroinflammation [34]. This finding suggests that inhibiting the infiltration of neutrophils and monocytes into the hippocampus may mitigate the development of spontaneous recurrent seizures induced by SE. As such, this strategy may offer a novel direction for epilepsy treatment.

The relationship between neuroinflammation and BBB leakage remains a problem - it is uncertain whether neuroinflammation is a causative factor, a consequence, or a co-instigator of BBB leakage. Alterations in the BBB may allow peripheral immune cells to access the CNS, subsequently activating CNS resident immune cells [35]. BBB damage does not require the involvement of peripheral immune cells, as reactive inflammatory astrocytes alone are sufficient to cause such damage [36].

### Innate Immunity Activation in Brain Resident Cells

Neuroinflammation is typically characterised by the activation of innate immune pathways within brain parenchymal cells. It is driven in epilepsy by cytokines and danger signals that activate downstream inflammatory signals such as complement and COXs [37]. Among the various contributors to the inflammatory response, neuroinflammation is a key component and may act as a driving factor in focal epilepsy. Microglia and astrocytes are key sources of inflammatory cytokines, danger signals, and downstream mediators in DRE, as evidenced by studies in human tissues and animal models of SE [38].

*Microglia*

Microglia, which constitute up to 10% of glia [39], are recognised as macrophage-like CNS resident immune cells [40]. The distribution of microglia is regionally specific, with the hippocampus, basal ganglia, and substantia nigra being the areas of the highest density [41]. In addition to influencing key developmental processes, including neuronal migration, white matter (WM) tract formation, vasculogenesis, and astrocyte formation [41], this localised concentration may be associated with the DRE pathological progression, such as hippocampal sclerosis. There are multiple ways by which microglia are activated from the resting state following CNS damage, including seizures. Such activations can be driven by cytokines, monocytes, neurotransmitters released by activated or damaged neurons, and molecules crossing the BBB [42]. Peripheral monocytic cells can interact with microglia by crossing the BBB or through neuronal networking [43].

An intriguing facet of microglial activation is its dichotomy, with two opposing states: the classical (M1) and alternative (M2) phenotypes [44]. The M1 microglia are traditionally considered to trigger inflammation (neurotoxin), while the M2 phenotype is primarily associated with suppressing inflammation (neuroprotective) [45]. Markers such as CD14, CD16, CD32, CD40, CD64, and CCL22 have been demonstrated to distinguish between the M1 and M2 phenotypes [46]. However, they fall short of accurately capturing the differentiation, activation, and functional states of microglia. Microglial activity is complex and subtly changes in response to local conditions, rendering the traditional M1/M2 or "good"/"bad" classification an oversimplification of its nuanced features.

These pro-inflammatory (M1) and anti-inflammatory (M2) states of microglia have implications for the pathogenesis of epilepsy. Pro-inflammatory cytokines such as TNFα released by M1 microglia can impair or alter the BBB, triggering a cascade of events [47]. This cascade includes neurogenesis, hyperexcitability, and decreased inhibitory neurotransmission. Generally, microglia promote neurogenesis and axonal sprouting through P2Y12 receptor signaling [48, 49]. They also limit excessive neurogenesis by phagocytosing ectopic newborn cells and inhibit axonal extension *via* IL-1B secretion [48, 49]. However, these regulatory functions become disrupted in the chronic neuroinflammation occurring in DRE [48]. Neuronal hyperexcitability is exacerbated by microglia-released TNF-α, amplifying neural circuit excitability [50]. In addition, microglia disrupt the excitatory-inhibitory balance in hippocampal circuits by selectively phagocytosing or stripping inhibitory inputs from nascent dentate granule cells [51]. Chronic neuronal network hyperexcitability lowers the seizure threshold, making the brain more susceptible to spontaneous recurrent seizures [52]. Earlier reports have linked elevated pro-inflammatory cytokines released by M1 microglia with increased frequency and total duration of seizures [53].

Whether microglia are destructive or beneficial for seizures depends on the stage of the disease or seizure activity [54]. While the post-seizure activation of microglia might contribute to neurodegeneration, their longer-term role may also be protective. This neuroprotective effect could result from the release of anti-inflammatory cytokines and the phagocytosis of apoptotic cell debris, indicating a switch to an M2 phenotype.

#### Astrocytes

The unique positioning of astrocytes between blood vessels and synapses allows them to manage synaptogenesis, carry out metabolic and homeostatic preservation duties, and maintain an ideal environment to ensure optimal neuronal function [55]. Increasing evidence suggests that astrocytes play an essential role in the pathophysiology of epilepsy [56]. Changes in astrocytes in response to environmental signals following epilepsy-induced brain injury include differences in morphology, biochemistry, and function. This process is called reactive astrogliosis [57]. Previous studies have confirmed that reactive astrocytes have the dual properties of impeding and promoting nervous system repair after injury [58]. Reactive astrocytes can be roughly classified into A1 and A2 types, each functioning differently [45, 59]. It is accepted that A1 astrocytes are destructive to neurons and synapses [60], while A2 astrocytes help maintain brain homeostasis by releasing neuroprotective cytokines [45, 60].

Astrocytes are closely linked to the pathogenesis of epilepsy [61]. Activated astrocytes may contribute to epilepsy *via* cytokine secretion, such as IL 21 in hippocampal tissue [62]. The activation of transient potential vanilloid receptor 1 on astrocyte membranes can promote the infiltration of pro-inflammatory factors into the vicinity of neurons by facilitating astrocyte migration, resulting in the high excitability of neurons and an accelerated seizure onset [63]. Targeting mTOR signalling in astrocytes can prevent the increased seizure frequency in temporal lobe epilepsy (TLE) [64].

Following brain injury, the normal communication patterns of the neurovascular unit at the BBB undergo significant alterations, with astrocytes primarily mediating the expression of tight junction proteins and the regulation of the extracellular environment [65]. Reactive astrocytes can remodel a damaged BBB and limit leukocyte infiltration from peripheral blood [66], but this is unlikely to outweigh the detrimental consequences of dysfunctional astrocytes. Bidirectional signalling between microglia and astrocytes deserves mention [67]. Activated microglia-triggered cytokines can transform naive astrocytes into A1-type reactive astrocytes, potentially influencing oligodendrocyte differentiation and survival through such glial interactions [59, 68]. So, microglia potentially affect the ability of astrocytes to preserve neuronal excitability. Activated astrocytes may also regulate microglia by releasing cytokines or chemokines in neuroinflammation [69, 70]. Astrocyte Cx can increase hippocampal microglial activation in DRE [71]. Microglia-astrocyte crosstalk may cause the perpetuation of a chronic inflammatory environment and contribute to the severity of seizures. In theory, inhibiting A1, reversing A1, and promoting A2 are beneficial and have great research potential. Regulating the interaction between microglia and astrocytes will provide new insights into treating epilepsy.

Evidence supports the role of neuron-astrocyte communication, which may affect DRE. Neuronal excitability can be altered through astrocytic Ca^2+^ signalling or regulated by the release of synaptically active gliotransmitters such as glutamate and ATP [72, 73]. An "astrocyte-neuron lactate shuttle" hypothesis suggests that lactate exported by astrocytes is the crucial energy source for neurons [74] in supporting glial cells. Glycogen deposited in astrocytes fuels neurons when extra energy is required during an epileptic seizure [75]. Thus, reducing cerebral glucose utilisation by interfering with glucose metabolism in astrocytes may have anticonvulsive effects. In addition, a neuroprotective property of gap junctions has been reported, and the therapeutic interference with Cx gap junction function may lead to side-effects [76]. Molecular agents inhibiting the Cx hemichannels but not Cx gap junctions may have important potential for antiepileptic therapies [11].

#### Oligodendrocytes

Within the WM, oligodendrocytes are widely associated with axons, enveloping neuronal membranes to generate myelin. Myelin loss and altered oligodendrocyte distribution are commonly reported in post-surgical tissues from various focal epilepsies and in their animal models [77]. The influence of microenvironmental remodelling in oligodendrogliomas on the emergence of DRE has yet to be fully understood. Notably, people with focal epilepsy triggered by oligodendrogliomas exhibit severe drug resistance [78]. Transcriptomic research has identified clusters marked by oligodendrocyte genes (MAG and MOG) within the pro-inflammatory microenvironment of epileptic tissue [79]. Epilepsy is closely associated with the death of oligodendrocytes and dysmyelination [80].

Recent animal work has uncovered significant demyelination changes in epileptic brains [81]. In the early stage of epilepsy, the production of numerous neurotrophic factors due to oligodendrocyte activation may limit the neural damage instigated by excitatory amino-acids, thus potentially averting the onset of epilepsy. In chronic epileptic conditions, alterations in oligodendrocyte proteins and a marked decrease in neurotrophic factors may exacerbate epilepsy [82, 83].

Monitoring the activation state and interaction of glial cells provides valuable insights into their impact on DRE progression. It also opens avenues for developing new therapeutic strategies to mitigate the onset and progression of epilepsy.

### Adaptive Immunity from Peripheral T and B Immune Cells

The activation of CNS-resident cells may be the primary driver of subsequent infiltration by peripheral immune cells in focal epilepsy. Acute brain injury triggers a rapid neuroinflammatory response in brain cells (within minutes to hours), followed by macrophage extravasation (over several days), with T cells appearing at later stages. In contrast, peripheral immune activation may act as the initial trigger in the autoimmune forms of epilepsy. Distinguishing the underlying causes of epilepsy is essential for understanding the immune microenvironments contributing to brain inflammation.

#### T Cells and Regulatory T Cells

Peripheral inflammation, driven by immune cells, can foster a low-grade inflammatory environment, leading to brain tissue damage with further generation of damage-associated molecular patterns (DAMPs). DAMPs can also be detected without adaptive immune cells, and danger signals are rapidly released during seizures, even without adaptive immune activation [84].

In response to this continuous pro-inflammatory milieu, there is a marked increase in IL-10 produced by regulatory T cells (Tregs; CD4+CD25+FoxP3+) in the peripheral blood of people with TLE [85]. The positive correlation between the frequency of Tregs and the age at onset of seizures has been highlighted [85]. A decline in activated and non-activated regulatory T cells in the peripheral blood of people with DRE compared to controls has also been reported [86]. These findings are further reinforced by evidence showing an inverse relationship between the number of regulatory Tregs and seizure severity in pediatric epilepsy, as observed in brain tissue [87]. The elevated Tregs might suggest a compensatory consequence to counteract the persistent pro-inflammatory stimuli. Conversely, a reduction in Tregs could signify a disruption in the intricate regulatory mechanisms, potentially leading to heightened drug-resistant inflammation. Studies on mice with SE have spotlighted the neuroprotective attributes of Tregs. Specifically, depleting autologous natural Tregs exacerbates, while supplementing them mitigates, seizure susceptibility in a kainic acid-induced mouse model [87].

Tregs's possible protective role offers promising avenues for innovative immunotherapeutic strategies targeting peripheral immune cells in epilepsy. The ketogenic diet has been shown to foster a Th17/Treg balance in the pathogenesis of DRE, primarily by inhibiting the mTOR/HIF-1α signalling pathway [88]. It is postulated that therapies capable of transitioning Th17 cells to Tregs, thereby achieving a balance during the initiation and progression of epilepsy, could serve as potent interventions.

#### B Cells and Regulatory B Cells

Autoimmune encephalitis (AIE), a neuroinflammatory disorder responsive to immunosuppressive therapy, is acknowledged as a potentially treatable aetiology of DRE [89]. A significant proportion of people with AIE develop DRE [90]. At the heart of AIE pathogenesis are B cells, activated by cytokines secreted by T cells [91]. The application of rituximab (RTX), an off-label B cell depletion therapy, has shown potential efficacy against intractable SE in autoimmune encephalitis [92]. As an anti-CD20 monoclonal antibody, RTX primarily targets memory and naïve B cells, sparing the long-lived plasma cells. This selective action leads to the persistence of pathogenic autoantibodies in some people with AIE, causing resistance to RTX [93]. Interestingly, a peripheral decrease in regulatory B cells (Bregs; CD20+/IL10) has been reported in people with temporal DRE [86]. In contrast to Tregs, considerably less is known about infiltrating Breg cells in seizures. Compared to neuro-inflammation driven by innate immune activation, the role of peripheral immune cells, particularly those of the adaptive immune system, in contributing to brain inflammation in epilepsy is relatively limited. However, Bregs, much like their T cell counterparts, may play a significant role in our understanding of immune responses in epilepsy and offer potential therapeutic targets for future interventions.

### Neuro-immune Interactions

Persuasive evidence has implicated neuro-immune interactions in epileptogenesis, as demonstrated by the infiltration of leukocytes in epilepsy-associated abnormalities, including neurodevelopmental aberrations [94]. The interactions between glial and immune cells are either direct or through inflammatory mediators. Previous studies have shown that the influx of Th17 cells into the CNS can further stimulate microglial activation, establishing a self-perpetuating cycle of inflammation and seizure activity [95]. Cutting-edge sequencing technologies indicate that, in surgically-resected epileptic zone tissue, an immune complex is directly assembled between T cells and microglia, facilitating the generation of the cytokine IL-1b and inciting inflammatory signalling [79]. Astrocytes engage in a dialogue with microglia and immune cells by mediating chemokines and cytokines, orchestrating the inflammatory response, and possibly manipulating the seizure threshold [12, 45]. Inflammatory cytokines such as IL-17, discharged by Th17 cells, can incite the astrocytic production of IL-6, a cytokine associated with enhanced neuronal excitability [96]. Astrocytes are among the initial CNS-resident cells that engage with the infiltrating immune cells. They govern the entry of monocytes, macrophages, and T cells into the CNS by releasing CCL2 and CXCL10 [97]. Astrocyte-produced CXCL12 can regulate the recruitment of pathogenic B-cells [98]. In addition, microglia can orchestrate the recruitment of monocytes and macrophages to the CNS *via* CXCR1-CCR2 and CCR2/CCR5 receptor-mediated pathways [31, 32, 86]. For oligodendrocytes, less studied in epilepsy, activated CD 4+ T cells can attack oligodendrocytes in neuroinflammation and destroy myelin structure, consequently impacting neuronal function [99]. Beyond glial cells, the interaction between neurons and immune cells also plays a crucial role in the pathophysiology of epilepsy. Neurons can directly interact with CD4+ T cells, leading to the differentiation and proliferation of Th1 and Th17 cell subgroups [100]. IL17 produced by Th17 cells can further activate microglia and increase neuronal excitability [101].

The role of neuroinflammation in DRE is gaining recognition, and one promising approach to managing DRE involves targeting these inflammatory pathways with targeted treatments. Small-molecule drugs targeting pro-inflammatory mediators such as IL-1β, IL6, TGF-β, TNF-α, HMGB1, CCL2, and COX-2 are becoming realistic candidates for inflammation in epilepsy [102]. A slew of preclinical investigations postulates that TLR4 or NF-κB inhibition can mitigate the frequency and duration of seizures, offering a potential therapeutic avenue for DRE [103–105]. Some individuals with Rasmussen's encephalitis (RE) showed seizure frequency improvement following anti-TNF-α therapy [106]. Inhibitors of the NLRP3 inflammasome, such as MCC950, have demonstrated encouraging results in preclinical models of epilepsy, suggesting their potential therapeutic utility in DRE management [107]. Anakinra, an IL-1 receptor antagonist, has shown potential in curtailing seizure frequency in animal models [108] and is presently undergoing scrutiny in clinical trials [109, 110]. Activated caspase-1 facilitates the proteolytic cleavage of the pro-inflammatory cytokines IL-1β and IL-18, and triggers an inflammatory form of programmed cell death known as pyroptosis [111]. The small molecule caspase-1 inhibitor CZL80 has been found to reduce seizures in models of pharmacoresistant TLE. In addition, it lowers neuronal excitability in brain slices from people with pharmacoresistant TLE [112]. The expression of caspase-3 is higher in the brain tissue of children and adults with DRE than in controls [113]. In summary, therapeutic strategies targeting caspase may offer a novel approach to alleviating neuroinflammation and enhancing the effectiveness of epilepsy treatment.

RE is a chronic inflammatory condition of the brain characterised by recurrent seizures that are often highly resistant to standard antiepileptic medications [114]. The hallmark histopathological feature of RE is inflammation targeting the CNS, characterised by T lymphocyte infiltration and microglial nodule formation, leading to significant neuronal loss and triggering neuronophagia and gliosis [115–117]. Immune suppression therapies, including anti-CD20, tacrolimus, intravenous Ig, and steroids, have achieved only moderate success in relieving RE [115–118]. A previous study has demonstrated that α4 integrin blockade effectively decreases CNS infiltration by immune peripheral cells. In this way, α4 integrin blockade significantly decreases the inflammatory markers linked to RE in the CNS, thereby reducing seizure frequency [119]. However, these findings were obtained using a humanised RE mouse model. Future clinical trials are necessary to validate the related research outcomes further.

Anti-inflammatory treatments may also modulate the structural and functional brain networks affected by DRE. Long-term administration of minocycline enhances neurogenesis and promotes the recovery of synaptic plasticity in epileptic rats, according to a report [120]. Anakinra has been shown to prevent neuronal loss in some hippocampus regions and has therapeutic potential in IL-1-driven systemic autoinflammation with refractory epilepsy [121]. More comprehensive clinical trials are warranted to test these interventions' effectiveness and safety thoroughly. These treatments act by modulating what we propose to call an "inflammation network" at the epicentre of glial cells in refractory SE. Systemic therapies can broadly modulate immune responses, but they may not effectively address local inflammatory changes and may even cause systemic side effects [97]. Developing more spatially-targeted immunomodulatory therapies can act more precisely on specific inflammatory regions, improve therapeutic effects, and reduce adverse reactions. Considering the spatial heterogeneity of inflammation, future research into more spatially-targeted immunomodulatory therapies will have significant clinical value.

We initially propose the concept of an "inflammation network" in refractory SE, which may serve as the epicentre of impending scholarly endeavours. This complex network, culminating in antiepileptic drug resistance, is shaped by the dynamic interplay between glial cells, neurons, invading immune cells, and diverse inflammatory molecules (see Table 1). In the pathogenesis of various brain diseases, inflammation networks are increasingly recognised as crucial components. In neurodegenerative diseases like Alzheimer's, the network activates microglia *via* amyloid-β, which releases pro-inflammatory cytokines and further neuronal damage [122]. Abnormal accumulation of α-synuclein activates microglia, triggering neuroinflammation and neurodegeneration in Parkinson's disease [123]. The inflammation network involves innate and adaptive immune responses in the context of cerebrovascular accidents or stroke [124]. Microglia are the primary macrophage-like cells in the CNS and serve as the first line of defence against stroke [124, 125]. In cerebral neoplasms such as glioblastoma, inflammation networks involve tumor cells, microglia, astrocytes, and infiltrating immune cells. These interactions promote tumor growth, invasiveness, and resistance to therapy [126]. Future disentanglement of these complex networks, including inflammation networks, will lead to novel therapeutic paths.

**Table 1:** The key immune interplay between glial cells and between glial cells and neurons

Interaction	Primary Targets	Possible Signaling Pathways	Major Function (Upon Overexpression or Activation)	Deterioration of the DRE	Potential Targeted Therapeutics
Microglia and Astrocytes	1. P2X7R [258]. 2. CCR2 [70]. 3. MFG-E8 [259] 4. C3aR [260, 261]	1. NLRP3 pathway. 2. CCL2/CCR2 axis. 3. NF-κB and PI3K-Akt pathways. 4. Classic cascade complement pathway.	1. Depolarization of the cell membrane, injury of neurons, synaptic reorganization, breakdown of the BBB, and lymphocyte accumulation. 2. Microglial activation, hippocampal neuronal apoptosis, reduction of synaptic proteins, and cognitive dysfunction. 3. Suppression of neurotoxic reactive astrocytes, suppression of inflammation, and neuroprotection. 4. Regulation of microglial phagocytosis, impairment of dendritic morphology and synaptic function, and cognitive dysfunction.	1. Increase 2. Possible increase 3. Possible decrease 4. Possible increase	1. JNJ-42253432, JNJ-47965567, AFC-5128. 2. RS504393. 3. None identified 4. C3aR antagonist.
Microglia and Oligodendrocytes	1. Galectin-3 [79, 262, 263] 2. TLRs [264] 3.PACAP/VIP [264]	1. BMP and Wnt signalling pathway. 2. NF-κB and PI3K-Akt pathways. 3. NF-κB and PI3K-Akt pathways, and induction of galectin-3.	1. Regulation of Microglial Phagocytosis of Myelin. 2. Engulfment of myelin debris facilitation. 3. Engulfment of myelin debris facilitation, neuroprotection, and blockage of microglia inflammation.	1. Uncertain 2. Uncertain 3. Possible decrease	1. None identified 2. None identified 3. None identified
Microglia and Neurons	1. P2Y12R [48] 2. CD39 [46, 265] 3. NMDAR [48] 4. CX3CR1 [266, 267] 5. CD200/CD200R [9]	1. ATP and purinergic signalling and the MAPK pathway. 2. CREB/CRTC1 signaling. 3. Glutamatergic signaling. 4. Fractalkine (CX3CR1–CX3CL1) signaling. 5. CD200 signalling.	1. Promotion of aberrant neurogenesis and immature neuronal projections following seizures. Regulation of synaptic plasticity, vascular repair, and microglial-neuronal physical interactions. 2. Maintenance of CNS homeostasis: suppression of epileptic neuronal hyperexcitability. 3. Downregulation of neuronal hyperactivity, neuroprotection. 4. Upregulation of neuronal excitability, microglial activation, neurodegeneration, and neuroblast formation. 5.Control of neuroinflammation, protecting neural functions.	1. Possible increase 2. Decrease 3. Decrease 4. Increase 5. Decrease	1. Clopidogrel. 2. None identified 3. AP5 (NMDA receptor antagonists). 4. Anti-FKN antibody. 5.Recombinant CD200 fragment crystallizable.
Astrocytes and Neurons	1. EAATs [268, 269] 2. GLUTs, MCTs [270–272] 3. GABA receptors, Glutamate receptors: NMDA, AMPA [273]	1. Glutamate-glutamine Cycle, NF-κB. 2. AMPK, Astrocyte-neuronal lactate shuttle. 3. Voltage-gated Na^+^, K^+^, and Ca^++^ channels; GABAergic and glutamatergic transmission.	1. Regulation of astrocyte glutamate uptake from the extracellular space in CNS: maintaining synaptic function and preventing neuronal damage due to excessive glutamate. 2. Modulation of astrocytic glycolysis: fueling neurons and bolstering neuronal operations. 3.Control of astrocyte glutamate secretion and paroxysmal depolarization shift.	1. Decrease 2. Decrease 3. Possible increase	1. EAAT Enhancers. 2. Ketogenic diet; β-hydroxybutyrate and acetoacetate. 3. Drugs suppressing glial calcium events.

(P2X7R, P2X7 Receptor; CCL2, C-C Motif Chemokine Ligand 2; NLRP3, NLR Family Pyrin Domain Containing 3; SAPK, Stress-Activated Protein Kinase; MAPK Mitogen-Activated Protein Kinase; MFG-E8, Milk Fat Globule Epidermal Growth Factor 8; CXC3L, C-X-C3 Motif Chemokine Ligand 1; CXC3R, C-X-C3 Chemokine Receptor 3; PI3K-Akt, Phosphatidylinositol 3-Kinase/Serine-Threonine Kinase; BMP, Bone Morphogenetic Protein; Wnt, Wingless/Integrated; TLRs, Toll-Like Receptors; PACAP, Pituitary Adenylate Cyclase-Activating Polypeptide; VIP, Vasoactive Intestinal Peptide; CREB/CRTC1, cAMP Responsive Element Binding Protein-Regulated Transcription Coactivator-1; ADAMT, A Disintegrin And Metalloproteinase With Thrombospondin Motifs; EAATs, Excitatory Amino Acid Transporters; GLT-1, Glutamate Transporter 1; GLUTs, Glucose Transporters; MCTs, Monocarboxylate Transporters; LDH, Lactate Dehydrogenase.)

## Ingenious Bridge: Forging a Nexus between Neuroinflammation and Brain Networks

The cornerstone of treatment for DRE is surgical intervention, whether through resection, minimally-invasive procedures, or neuromodulation. Preoperative planning requires the identification of the site of ictogenesis. The epileptogenic zone (EZ) is defined as "the brain region indispensable for seizure generation, whose removal or disconnection is necessary for seizure cessation." The EZ encompasses the seizure onset zone (SOZ) or is located within it [127]. However, the EZ cannot be identified in some people, making them ineligible for surgical interventions [128]. In addition, an individual may have multiple independent EZs. In some cases, new EZs may emerge after the initial EZ is removed [129]. This variability complicates the prediction of surgical outcomes based solely on EZ identification. The epilepsy network theory proposes that the EZ is extensively connected to other brain regions, and seizures can originate from any part of the network. The network theory provides a comprehensive framework for predicting surgical outcomes [127].

Epilepsy is characterized by disruptions in connectivity, function, and neurobiology, such as the presence of grey matter heterotopias, WM alterations, and cortical folding [130–132], which tend to follow identifiable network-like patterns [133]. These alterations in brain network architectures serve as the foundation for aberrant functional networks, which we call the "epileptogenic network". The comprehensive manifestation of seizure symptoms in individuals afflicted with DRE arises from the propagation or oscillation of discharges within this epileptogenic network [134, 135], rather than solely being attributed to bursts confined to a particular focus. It is important to note that no singular cortical area possesses autonomous functionality; rather, the realization of distinct functions stems from the dynamic interplay of functional connectivity (FC) between diverse brain regions [136]. Focal seizures transcend the notion of mere focal involvement, encompassing many cerebral regions that need not be confined to immediate anatomical proximity [137, 138].

Brain networks are often discussed in terms of graph theory and connectivity as mathematical concepts, but their relationships with inflammation and the neurovascular unit are increasingly being recognized. As a result, information from macroscopic and microscopic levels is being integrated through mesoscopic scales, such as the connectivity patterns between neurons and the functional networks of specific brain regions. IL-6 has been linked to brain structure and may influence regions involved in developmental neuropsychiatric disorders, such as schizophrenia and autism [139]. Such a process instigates a cascade of inflammatory responses that engender alterations in cerebral morphology by eliciting microglial activation and/or astrocytic dysfunction [139]. For example, the spread of Alzheimer's disease pathology is believed to occur through WM fiber connections, involving the trans-synaptic transmission of toxic proteins along neuronal pathways. However, research indicates that despite the loss of WM tract integrity, pathological neural networks can still mediate the spread of pathology within the Alzheimer's disease spectrum [140]. This begs the question of whether the generation and dissemination of neuroinflammation also adhere to underlying network patterns. Exploring the role of neuroinflammation in the development of DRE and related aspects through the utilization of specific network biomarkers or analytical methods appears to hold promise. Addressing network abnormalities in DRE caused by the synchronous effects of over-excited neurons, activated glial cells, oxidative stress, and neuroinflammation requires comprehensive strategies based on molecular biology, structural neuroimaging, and bioengineering.

### Imaging and Analytical Methods for Brain Network Abnormalities in Epilepsy

#### MRI-Based Techniques

MRI remains a mainstay in epilepsy research, with developments in functional MRI (fMRI) enabling the exploration of network dynamics in real time [141]. Structural MRI (T1-weighted and FLAIR imaging) is one of the essential modalities in the presurgical evaluation of DRE [142]. It allows for non-invasive lesion detection and helps define surgical targets in most people, making it indispensable in presurgical workups [142]. The presence of a lesion on structural MRI is by far the most critical predictor of seizure-free outcome after DRE surgery. Further advancing our understanding of epileptic networks, diffusion tensor imaging (DTI) has shed light on WM integrity and connectivity [143]. MRI-based techniques are also expected to open avenues for exploring neuroinflammation. One of the potential focal points is the glymphatic network, the brain's pseudo-lymphatic waste-clearance system [144, 145]. Its structure and function are impaired in neuroinflammatory conditions characterized by adaptive immune cell infiltration [146, 147]. Magnetic resonance encephalography and DTI analysis along the perivascular space have been introduced to assess glymphatic function [148, 149]. Magnetic resonance spectroscopy (MRS) is also a promising tool for specifically detecting glial cell changes associated with neuroinflammation. MRS shows sensitivity in detecting microglial reactivity across populations with varying levels of neuroinflammation and has significant potential for broad applications in research and clinical settings [150]. This represents an exciting step towards finding an imaging-based biomarker of glymphatic function. The techniques and conceptual basis of studying the glymphatic system may apply to the study of epilepsy. Integrating multimodal MRI with macroscale connectomics holds promise for better understanding the network and inflammatory features of DREs [151].

#### PET/SPECT

PET/SPECT is optimally used for metabolic network imaging using radiotracers, providing unique insights into the neurochemical processes associated with epilepsy [152–154]. These modalities complement the structural and functional network information gleaned from MRI. Radioligands targeting translocator protein (TSPO), an indicator of activated microglia and astrocytes, provide a means to visualize neuroinflammation *in vivo* [155]. Increased TSPO binding in the epileptogenic zone has been found in people with DRE [156]. TSPO PET imaging has shown promise in studying neuroinflammation, but it is important to note that this method does not work for all participants due to genetic variability [156, 157]. Specifically, a polymorphism in the TSPO gene affects the binding affinity of second-generation TSPO tracers, which requires genetic screening prior to use to ensure accurate results [157]. 11C-α-methyl-l-tryptophan PET allows visualization of the inflammatory kynurenine pathway to epileptogenesis [158]. In a separate but related vein, evidence suggests that drug resistance in epilepsy might stem from abnormally upregulated Pgp efflux transporters at the BBB [159]. This has led to a novel approach to imaging the BBB with Pgp-based PET, followed by targeted therapy to overcome Pgp over-activity. In addition, deprenyl, as a glial cell tracer, can be used in combination with PET to detect reactive astrogliosis under various neuroinflammatory conditions [160]. These approaches contribute to the neuroimaging of metabolic networks in epilepsy, and the choice of technique can be tailored based on the specific target compound.

#### EEG/MEG

An array of non-invasive modalities, encompassing MRI, PET/SPECT, and EEG/MEG, can proffer independent information on the location of the presumptive EZ. The superior temporal precision of EEG and MEG facilitates the capture of transient network dynamics allied with epileptic activity [161–163]. Applying dynamical network models to MEG/EEG data to identify the EZ is a promising advance [164, 165]. As a rule, network modeling mandates a connectivity analysis to obtain a network structure or topology [165]. As examples of such connectivity analyses, ictal imaging using electrical source imaging from scalp EEG, in tandem with FC analysis, considers not merely the most active source [166] but also the magnitude of its signal propagation to other cerebral areas, known as outgoing connections [167]. While scalp EEG offers valuable insights, intracranial EEG such as SEEG and electrocorticography provides a high-resolution view of internal brain activity with superior signal clarity and precise epileptic focus localization, crucial for identifying complex seizure patterns [168]. They are typically used as a preparatory step before surgical resection or ablation of the epileptogenic focus. Intracranial EEG, integrated with artificial intelligence technologies such as deep learning, can detect subtle changes in frequency and amplitude, thereby advancing the development of sophisticated diagnostic models that improve seizure prediction and classification [169]. Incorporating source localization techniques has augmented the spatial resolution of these modalities, making them even more potent tools in the fight against epilepsy [162]. Subsequent research endeavors will need to find optimal approaches for measuring the strength of connectivity and the direction of information transmission within neuronal networks.

Besides, advanced computational techniques like graph theory have revolutionized how we interpret these imaging data, allowing for the characterization of brain networks in a more data-driven and holistic manner [170]. When coupled with machine learning methodologies, these techniques augur well for the automation of seizure detection and prediction [171, 172]. It is feasible to envisage a network system of DRE where structural details from diffusion MRI/structural MRI/DTI, functional readings from fMRI/EEG/MEG, along with metabolic and immune profiles gleaned from PET/SPECT [173–178], are consolidated through the prism of graph theory and machine learning. This would give rise to comprehensive 'brain maps' that offer a panoramic view of the brain's various facets.

### Locations and Characteristics of Brain Network Abnormalities

The abnormal neural network in individuals afflicted with mesial temporal lobe epilepsy (MTLE) extends far beyond the boundaries of medial temporal lobe structures [179]. The crucial functional connectivity between mesial temporal and default mode structures is paramount in meticulously planning surgical interventions [131, 180]. This section illuminates the localization of the aberrant brain network implicated in DRE, elucidates its excitatory and inhibitory attributes, and discerns its intricate association with neuro-inflammation.

#### Structural Networks and Neuroinflammation

In a comprehensive context, the structural networks encompass molecular biology and computer science interpretations, a paradigm equally applicable to functional networks within the brain. Structural DRE networks are defined by structural connectivity grounded in anatomy and molecular biology. Network abnormalities in epilepsy are extensive and multifaceted. Taking MTLE as an example, it primarily presents itself as abnormal epileptic networks between structures like hippocampal sclerosis or mesial temporal regions and other regions associated with epilepsy (such as the limbic system, thalamus, frontal and motor regions) [181–183]. The alterations in the topological arrangement of networks in TLE are manifested explicitly in the reduced cortical/subcortical connectivity with increased clustering and path length in temporal and orbitofrontal regions [178, 184], indicating a shift towards network regularization. The contralateral hemisphere shows a compensatory reorganization and striking reconfigurations of large‐scale networks [185]. Structural networks are also believed to be fast-evolving dynamical networks because such extensive structural abnormalities might not be independent but potentially interact with other pathological progress, among which neuroinflammation could be a key player.

Inflammatory cascades, initiated by factors like cytokines, chemokines, and glial activation, can trigger neuronal loss and aberrant synaptic remodeling [37]. Prolonged seizure activity induces notable neuroinflammation, disrupting the brain's neuronal density and typical architecture, and pushing the structural network toward a state of dysregulation [186, 187]. People with DRE have significantly greater extra-axonal diffusivity markers than those with nonrefractory epilepsy; these alterations can be markers of neuroinflammatory processes [188]. Hippocampal atrophy and diffusivity changes in hippocampal white and grey matter have been reported following experimentally induced neuroinflammation *via* lipopolysaccharide administration [189]. *In vivo* and *in vitro* models of epilepsy provide further substantiation of this interaction [190–193]. The structural changes induced by inflammation also disrupt the balance between excitation and inhibition, a cornerstone of normal brain function, further perpetuating the cycle of seizures in epilepsy [186, 194, 195]. Overall, this inflammation-driven structural alteration, in essence, transforms the brain's network topology, pushing it towards a 'hyper-connected state' that facilitates the propagation of epileptic activity.

#### Functional Networks and Neuroinflammation

Functional brain networks reflect ongoing, synchronous neuronal activity across various regions [196]. When significantly disrupted, the topological organization of structural networks can result in abnormalities in their functional properties [197]. These disruptions have the potential to contribute to a susceptibility to seizure generation. Assessing the interplay between structure and function, the superficial WM emerges as a critical candidate region [198]. In people with TLE, there is a decrease in FCs between the superficial WM and the ipsilateral hippocampus (grey matter foci), implying an inadequate functional integration in unilateral TLE [199]. This may trigger a compensatory increase in FCs within the contralateral hippocampus and associated networks. Analysis of SEEG changes at the onset of temporal lobe seizures indicates frequent functional connectivity within the amygdala–hippocampus–entorhinal cortex network, alongside a concurrent absence of coupling with neocortical structures [200]. In addition, there is an increase in local efficiency within networks, evident as reinforced FCs within the epileptogenic zone and propagation zone [137, 201], suggesting more diffuse disease and a poorer post-surgical prognosis. Due to the heterogeneous hemispheric lateralization effect in functional networks not being clearly explained, the pattern of functional networks between the right TLE and left TLE is controversial [180, 202, 203].

The role of inflammation in shaping the FC landscape in epilepsy is expected to be a crucial aspect of understanding the disease. Brain regions with heightened inflammatory activity, identified through PET imaging, exhibit altered FC patterns, indicating the reciprocity between inflammatory and functional networks [204, 205]. Inflammatory mediators can modulate synaptic transmission, alter neuronal excitability, and promote aberrant network synchronization, redefining the FC landscape [206, 207]. For instance, IL-1β obstructs γ-aminobutyric acid (GABA)-mediated neurotransmission, restricts astrocyte glutamate uptake, and adjusts neuronal arousal [208]. In addition, the overexpression of IL-6 in the CNS induces abnormal hippocampal arousal, unprovoked seizures, and neurodegeneration [209]. Evidence supports this association, as individuals exhibit elevated levels of pro-inflammatory cytokines, which correlate with increased seizure susceptibility, cognitive impairment, and neuropsychiatric disorders [209, 210]. Cerebral hypometabolism may also highlight the development of aberrant glucose metabolism, which ultimately contributes to epileptogenesis [211], while neuroinflammation can induce abnormalities of cerebral metabolism in people with DRE [37]. These understandings make anti-inflammatory treatments a promising therapeutic avenue, as they have shown the potential to modulate the activity and connectivity of functional networks and improve clinical outcomes [212, 213].

**Fig. 2:**
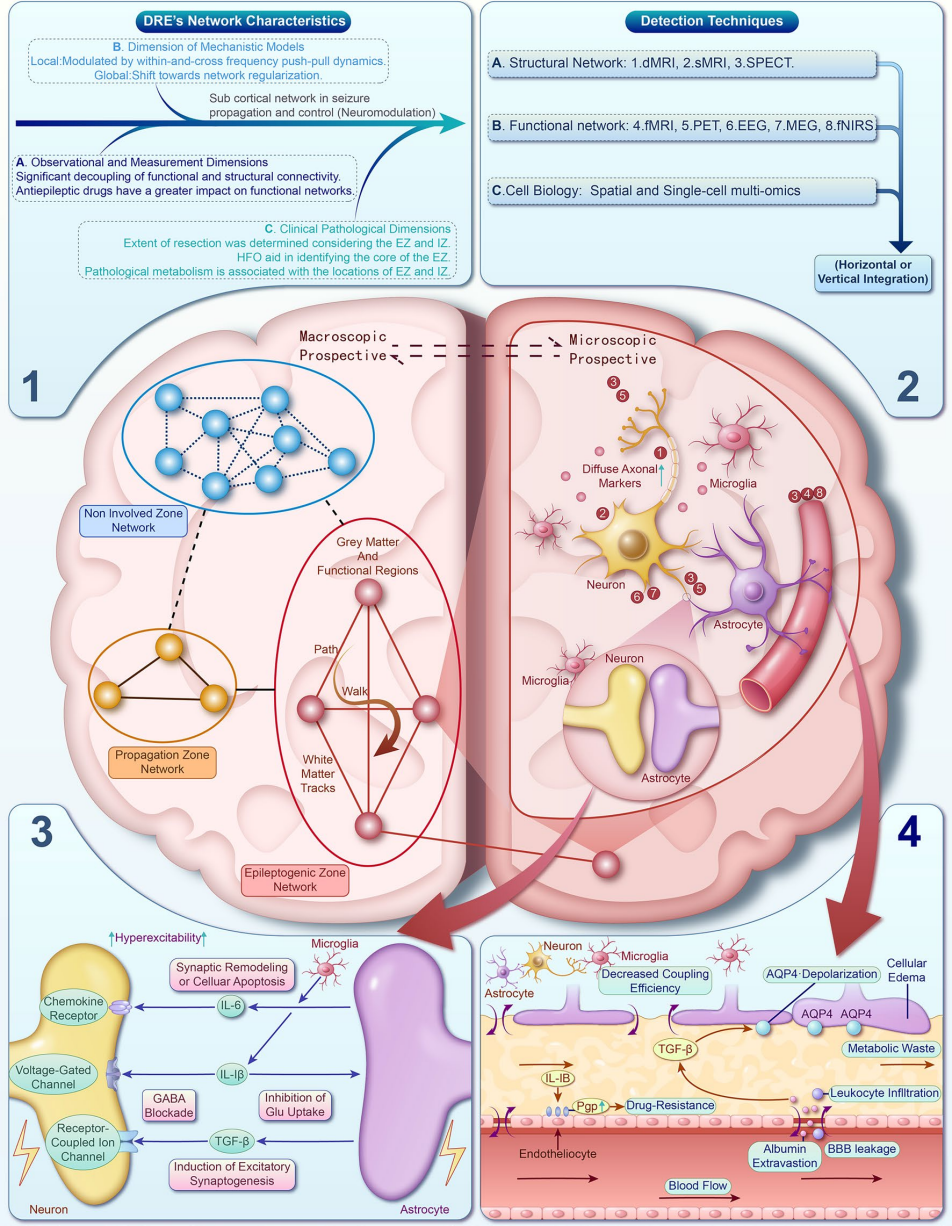
The landscape of the epileptic brain network. Epilepsy manifests as a network disorder, with the EZ serving as the focal point for seizure initiation. Beyond the EZ, seizures propagate through a distinct propagation zone network, complicating the simplistic model of a singular epileptogenic focus. Meanwhile, non-involved zone networks remain functionally insulated from seizure activity, often serving as surgical sanctuaries. The challenge lies in surgical planning, where the EZ may overlap with functionally critical regions. Advanced computational models allow for personalised surgical strategies, optimising the balance between seizure control and functional preservation. These models leverage structural connectivity data to identify optimal targets for resection or disconnection, offering a nuanced approach to surgical intervention. **Part 1** The three dimensions of DRE showing a decoupling of functional and structural connectivity, influenced by antiepileptic drugs. Mechanistically, it exhibits local push-pull dynamics and global network regularization. Clinically, resection surgery and neuromodulation are guided by the epileptogenic and irritative zones, with high-frequency oscillations and metabolic markers aiding in their identification. **Part 2** Besides mainstream techniques for assessing structural and functional networks (with target sites in figures), complementary approaches can be integrated with spatial and single-cell muti-omics. This will provide multi-faceted, in-depth insights into the inflammatory networks in DRE. **Part 3** Inflammatory factors such as IL-1β, IL-6, and TGF-β, released by microglia and astrocytes, disrupt GABA metabolism and neurotransmission, leading to abnormal neuronal arousal, increased seizure susceptibility, and excitatory synaptogenesis. **Part 4** Epilepsy-induced BBB disruption leads to albumin leakage, triggering TGF-β hyperactivation and cellular edema. This cascade of pro-inflammatory responses is primarily modulated by interactions with peripheral immune cells, astrocytes, and microglia. (EZ, Epileptogenic Zone; DRE, Drug-resistant Epilepsy; dMRI, Diffusion Magnetic Resonance Imaging; sMRI, Structural Magnetic Resonance Imaging; SPECT, Single Photon Emission Computed Tomography; fMRI, Functional Magnetic Resonance Imaging; PET, Positron Emission Tomography; EEG, Electroencephalogram; MEG, Magnetoencephalography; fNIRS, Functional Near-Infrared Spectroscopy; IL, Interleukin; TGF-β, Transforming Growth Factor Beta; GABA, Gamma-Aminobutyric Acid; BBB, Blood-Brain Barrier).

Neuroinflammation, along with structural and functional network aberrations, co-exist and interact in epilepsy, each shaping the other in a complex, dynamic interplay (Fig. 2). Understanding this relationship is vital for developing therapeutic strategies targeting the inflammatory component and its consequences for functional connectivity.

#### Unravelling the "Inflammation Network" in DRE

Incorporating the "Inflammation Network" concept in epilepsy involves understanding the networked nature of neuroinflammation, its diffusion characteristics, and its intricate interactions with the structural and functional brain networks in DRE. The function of this network includes direct actions of inflammatory molecules on neurons or mediated by inflammatory glia-neuron communication. Neuroinflammation in epilepsy is a dispersed event [214]; the inflammatory process extends beyond the epileptogenic zone [215], suggesting an intricate network of inflammatory responses across the brain. For example, inflammatory mediators and activated microglia show distinctive spatial distributions and levels of expression following epileptic seizures [216–218], evident in functionally interconnected brain regions [218]. Considering temporal aspects, the phenotypic transition of microglia into pro- or anti-inflammatory forms across different phases of DRE is key [219, 220], along with interaction with other neuroglia, which significantly influences CNS immune cell recruitment, inflammatory response initiation, chronic demyelination, and axonal loss [221]. The exact characteristics of inflammatory networks' spatial and temporal distribution warrant further exploration.

The propagation of the inflammation network in DRE may follow specific routes, possibly around the perivascular spaces and the parenchyma, where seizure onset commonly occurs [194]. The spread of inflammation is typically associated with the proximity of inflammatory cells and the inflammatory microenvironment, but inflammatory mediators can spread through the blood or cerebrospinal fluid in the brain, affecting areas far from the initial source of inflammation. The cascade triggered by seizures, such as microglial activation and cytokine release [222], may compromise BBB integrity [26, 102], thus potentially facilitating the spread of inflammation. Besides, alterations in BBB permeability may stimulate the expression of inflammatory molecules in astrocytes [223]. This recurring cycle catalyzes the perpetuation of seizures and promotes cellular loss, contributing to maladaptive plasticity in neural networks. In conjunction with this, the structure and distribution of pericytes, vital constituents of the BBB, undergo significant changes as seizures progress [28, 224]. Given their pronounced sensitivity to microglial and inflammatory mediators [224], these transformations are likely substantial indicators of the propagation pathway for neuroinflammation in the epileptic landscape.

The epilepsy inflammatory network showcases a dynamic and extensive interplay of neuroinflammation within functional and structural brain networks in DRE, indicating a nuanced landscape of inflammation propagation facilitated by disruption of BBB integrity and spatiotemporal changes in glia. These factors are pivotal in perpetuating seizures and inducing maladaptive neural plasticity. We are only beginning to unravel this complex "Inflammation Network" in epilepsy, and much remains unknown about its architecture, diffusion patterns, interactions with the epileptic network, and consequential implications for epilepsy management. Utilizing fMRI in conjunction with PET may offer a more comprehensive perspective for exploring the relationship between epilepsy and inflammation.

### Emerging Treatments Using Brain Network Abnormalities as a Breakthrough

#### Surgery

Surgical resection of the epileptogenic focus remains the most effective intervention for controlling seizure frequency. Various surgical approaches, such as anterior temporal lobectomy and selective amygdalohippocampectomy, offer curative potential but are limited to specific individuals with well-localized seizure foci [225]. The success of surgical interventions hinges on the precise identification of the SOZ. Accurately localizing the SOZ allows physicians to target and remove or disrupt the region responsible for abnormal activity, effectively reducing seizure frequency and severity. Furthermore, identifying the SOZ helps minimize damage to healthy brain tissue, preserving neurological function and cognitive abilities as much as possible.

However, this task is challenging due to the complexity of brain networks and the variability in epilepsy presentation [226]. The SOZ is composed of multiple interconnected components with dynamic connectivity, making it difficult to achieve complete disruption. Following the resection or disconnection of the SOZ, additional onset zones that are more difficult to detect—due to factors such as depth, focality, eloquence, lateralization, and myelination—may still persist [227]. Thus, one of the key challenges in defining and disrupting the epileptogenic network is its reorganization over time. The dynamic characteristics of epilepsy networks appear to vary depending on the type of seizure and syndrome [228]. For example, people with Rasmussen encephalitis typically develop a rapidly-expanding seizure network as the disease progresses, leading to worsening epilepsy and neurological decline [118]. As a result, the most effective strategy may involve disrupting the global network, given the ongoing challenges in accurately defining and structurally disrupting the electrical connectivity. Moreover, open surgeries carry a significantly higher risk of postoperative neurocognitive impairments, such as optic nerve damage and cognitive dysfunction.

#### Brain-Computer Interface Technology

Intersectional advances in neural decoding and neuromodulation have led to innovative technologies, such as BCIs, that are based on neuroimaging. These have allowed us to glimpse the depths of the "deep brain" [229] and herald a promising option for people with DRE. BCIs function through direct communication between the brain and an external device. They record electrical activity from the brain, typically through EEG (intra/extracranial), and convert these signals into commands that control external devices or feed back into the nervous system [230]. This technology's direct interaction with the brain network allows the identification of seizure precursors and network abnormalities, providing a foundation for closed-loop neuromodulation [231].

#### Neuromodulation Methods

Empirical evidence substantiates that various neuromodulation therapies may effectively modulate inflammation within cerebral regions (Table 2). Neuromodulatory therapies treat DRE by directly altering the excitability of affected brain circuits. The mechanisms contributing to long-term improvement are not yet fully understood but may involve neuroinflammation, upregulation of GABA function, chronic glial changes, gene induction, and neurogenesis. The therapeutic effectiveness of neuromodulation primarily derives from its capacity to desynchronise epileptogenic neural networks. Deep brain stimulation (DBS), vagus nerve stimulation (VNS), and responsive neurostimulation (RNS) are mainstream neuromodulatory technologies approved by the US Food and Drug Administration (FDA) for treating DRE.

**Table 2:** Major neuromodulation therapies based on the brain network in DRE

Neuromodulation Technique	Underlying Mechanism for Epilepsy Treatment	Impact on Inflammation	Safety
DBS [232, 236, 237]	DBS primarily targets the ANT, CMT, and hippocampus. Its efficacy in epilepsy is thought to stem from disrupting neural synchronization within the Papez circuit and epileptic networks. While ANT and hippocampal targets are mainly used for focal epilepsy, CMT has shown effectiveness in treating generalized epilepsy.	DBS may have anti-inflammatory and anti-apoptotic effects in epilepsy, specifically targeting IL-1β and IL-6 cytokines. It attenuates CX3CL1/CX3CR1 signaling, inhibits microglial activation, and suppresses NF-κB expression when applied to the subthalamic nucleus.	DBS poses risks of implant site infection at hardware removal, and stimulation-related mood and cognitive disturbances. Few people report depression and memory issues, and these effects may improve over time; however, acute cognitive alterations can occur.
VNS [240–242]	The efficacy of VNS is attributed to immediate and sustained neuromodulation of critical neural pathways, encompassing the brainstem, thalamus, and cortex. It mainly targets catecholaminergic nuclei and limbic structures. Recent advances have introduced transcutaneous alternatives.	VNS has anti-inflammatory effects *via* the parasympathetic anti-inflammatory pathway, attenuating peripheral cytokine levels and central microglial activation. It targets the A7 subunit of the nicotinic acetylcholine receptor, which is crucial for cytokine regulation.	Surgical complications of VNS are uncommon but include site infection and vocal cord issues. Most side effects, like hoarseness, are transient and adjustable. Sleep breathing issues may worsen but are generally manageable.
RNS [243, 244]	The RNS system employs a closed-loop approach to dynamically and continuously monitor intracranial EEG at suspected seizure onset zones. Upon identifying individualized ictal patterns, it delivers targeted high-frequency electrical bursts to preemptively terminate potential seizures. Its real-time capabilities allow for adaptive, precise treatment capable of simultaneously addressing multiple epileptogenic foci.	Current evidence is scant regarding the impact of RNS on inflammatory activity in DRE or other neuropsychiatric disorders.	A minor risk of infection exists in individuals receiving RNS implants, occasionally leading to device removal but rarely causing severe neurological conditions. Some experience haemorrhage or suicidal ideation, while long-term follow-up generally shows cognitive stability or even improvement in specific domains.

(DBS, Deep Brain Stimulation; ANT, anterior nucleus of the thalamus; CMT, centromedian thalamic nucleus; IL, Interleukin; CXC3L, C-X-C3 Motif Chemokine Ligand 1; CXC3R, C-X-C3 Chemokine Receptor 3; VSN, Vagus Nerve Stimulation; RNS, Responsive Neurostimulation; TES, Transcranial Electrical Stimulation; EEG, electroencephalogram.)

DBS involves sending electrical impulses to specific brain areas involved in seizure initiation and propagation, such as the anterior nucleus of the thalamus [232]. DBS can directly interact with the brain's network and inhibit the abnormal neuronal activity underlying seizures [232]. Clinical trials have demonstrated DBS efficacy in reducing seizure frequency in some people with DRE [233]. While research suggests that DBS can potentially instigate neuroinflammation, characterized by astrocyte and microglial activation [234, 235], it's plausible that such outcomes stem primarily from electrode placement. Conversely, DBS may have potent anti-inflammatory impacts in epilepsy, influencing specific cytokines [236]. Application of DBS in the subthalamic nucleus has been found to diminish CX3CL1/CX3CR1 signalling and suppress microglial activation and NF-κB expression, ultimately leading to a consequential reduction in pro-inflammatory cytokines, specifically IL-1β and IL-6 [237].

VNS engages the vagus nerve through electrical stimulation, embodying a complex neuroendocrine-immune system. This interconnected network, crucial in maintaining homeostasis, establishes a pivotal link between the brain and numerous organ systems [238]. Clinical research has shown VNS to reduce the frequency and intensity of seizures, likely due to its broad influence over the brain's networks [239]. VNS diminishes the inflammatory response peripherally, through cytokine modifications, and centrally, by limiting microglial activation, a process known as the parasympathetic anti-inflammatory pathway [240]. The excitation of the parasympathetic system and subsequent release of acetylcholine stimulate the A7 subunit of the nicotinic acetylcholine receptor [241, 242]. This interaction is critical in controlling cytokine transcription and translation, having anti-inflammatory effects. Currently, non-invasive VNS techniques, whether percutaneous or transaural, may rival invasive direct stimulation's effectiveness in treating DRE.

RNS operates as a closed-loop system, detecting abnormal neuronal activity and responding with electrical stimulation to inhibit seizures [243]. Theoretically, this approach allows closed-loop RNS to incorporate sensing and stimulation electrodes within the SOZ. This potential action might trigger plastic alterations within the SOZ, which could diminish the incidence of focal-onset seizures [244]. With its capacity for real-time responses to evolving network dynamics, clinical studies have shown that RNS can substantially reduce seizure frequency, improving the quality of life for individuals [244, 245]. Despite the promising outcomes of RNS, one crucial aspect remains under-explored. There is a shortage of evidence addressing its role in neuroinflammation, which may offer significant insights for future research.

In addition to FDA-approved neuromodulation therapies for DRE, an expanding area of research is investigating alternative neuromodulation strategies with promising therapeutic potential [246]. By attenuating hyperactivity in epileptogenic zones or key relay structures like the thalamus, tDCS may reduce seizure propensity [247]. tDCS modulates neuroinflammation and activates endogenous neural stem cells post-stroke [248]. TMS applies a magnetic field to alter neuronal activity in local superficial areas of the brain. Low-frequency TMS dampens astrocyte-mediated inflammation, while high-frequency TMS reduces pro-inflammatory TNF-α and boosts anti-inflammatory IL-10 [249]. Intermittent θ-burst TMS further modulates microglial polarization, exhibiting an anti-inflammatory effect [249].

#### Personalization and Future Prospects of Neuromodulation

Current neuromodulation therapies are primarily palliative [250, 251]. Personalized closed-loop therapies with embedded machine learning, where the target and stimulation parameters are tailored based on individual brain network characteristics, could enhance therapeutic outcomes. To achieve personalised targeting and parameter settings in closed-loop neuromodulation, it is essential to conduct clinical mapping, detect biomarkers, and effectively assess network and structural connectivity and the feasibility of closed-loop control [252]. Personalised neuromodulation starts with clinical mapping. This process determines potential sites where stimulation consistently leads to symptom improvement. Various doses and different symptom severity levels are considered to ensure effectiveness. Candidate sensing locations were identified by a comprehensive correlation of neural activity with symptom severity, thereby identifying disease-related biomarkers. Effective network connectivity was evaluated by analysing the responses evoked at nodes across the network when single-pulse stimulation was applied to candidate targets. Structural connectivity between candidate contact pairs was assessed using tractography. Lastly, the feasibility of closed-loop control was evaluated by analyzing how stimulation at potential sites affected the identified biomarkers in candidate sensing locations.

Advances in artificial intelligence have also improved seizure prediction from EEG data, enhancing the efficacy of closed-loop systems [253, 254]. However, certain obstacles, such as the immune response in neural tissue and limited data processing capacity, have hampered the clinical translation of neuronal devices. We believe that many neurotechnologies (exemplified by BCIs), combined with anti-inflammatory therapies, hold promise for improving the well-being of people with DRE and those afflicted by other neurological disorders. They may also further advance our understanding of neural networks and brain function.

## Conclusion

Neuroinflammation seems to be a pivotal element in the genesis of DRE and provides fertile terrain for exploration. The multifaceted interplay among different peripheral immune cells, neuroglia, and neurons gives rise to what can be termed an 'Inflammatory Network'. This complex construct precipitates the dysregulation of the endogenous antiepileptic system, culminating in epileptic paroxysms. This inflammatory nexus's incessant perturbation of cerebral homeostasis is inherently intertwined with its synergistic intercommunication with structural and functional neural networks. In the spatial context, the propagation of this inflammatory web appears to be linked with the cerebral microvasculature and lymphatic architecture, exhibiting distinct patterns across various brain territories; in the temporal dimension, the phenotypic polarisation of microglial cells emerges as a cardinal component within this inflammation network.

Concurrent with the progressive strides in neuroimaging and neural networks, the amalgamation of neuroinflammation and brain network dynamics is expected to transcend the theoretical and enter the practical realm. Anchored in advanced neuromodulation methodologies and different stages of the onset of DRE, targeted interventions directed at peripheral immune cells and glial structures may well herald a new epoch of therapeutic avenues for those grappling with this condition.

## References

[1] Ding D, Zhou D, Sander JW, Wang W, Li S, Hong Z. Epilepsy in China: Major progress in the past two decades. Lancet Neurol 2021, 20: 316–326. 33743240 10.1016/S1474-4422(21)00023-5

[2] Perucca E, Perucca P, White HS, Wirrell EC. Drug resistance in epilepsy. Lancet Neurol 2023, 22: 723–734. 37352888 10.1016/S1474-4422(23)00151-5

[3] Amanollahi M, Jameie M, Heidari A, Rezaei N. The dialogue between neuroinflammation and adult neurogenesis: Mechanisms involved and alterations in neurological diseases. Mol Neurobiol 2023, 60: 923–959. 36383328 10.1007/s12035-022-03102-z

[4] Yang T, Velagapudi R, Terrando N. Neuroinflammation after surgery: From mechanisms to therapeutic targets. Nat Immunol 2020, 21: 1319–1326. 33077953 10.1038/s41590-020-00812-1 PMC7704062

[5] Vezzani A, Di Sapia R, Kebede V, Balosso S, Ravizza T. Neuroimmunology of status epilepticus. Epilepsy Behav 2023, 140: 109095. 36753859 10.1016/j.yebeh.2023.109095

[6] Ammothumkandy A, Ravina K, Wolseley V, Tartt AN, Yu PN, Corona L. Altered adult neurogenesis and gliogenesis in patients with mesial temporal lobe epilepsy. Nat Neurosci 2022, 25: 493–503. 35383330 10.1038/s41593-022-01044-2 PMC9097543

[7] Lentini C, d’Orange M, Marichal N, Trottmann MM, Vignoles R, Foucault L, *et al*. Reprogramming reactive glia into interneurons reduces chronic seizure activity in a mouse model of mesial temporal lobe epilepsy. Cell Stem Cell 2021, 28: 2104-2121.e10. 34592167 10.1016/j.stem.2021.09.002 PMC8657801

[8] Chen ZP, Wang S, Zhao X, Fang W, Wang Z, Ye H, *et al*. Lipid-accumulated reactive astrocytes promote disease progression in epilepsy. Nat Neurosci 2023, 26: 542–554. 36941428 10.1038/s41593-023-01288-6

[9] Yu C, Deng XJ, Xu D. Microglia in epilepsy. Neurobiol Dis 2023, 185: 106249. 37536386 10.1016/j.nbd.2023.106249

[10] Andoh M, Ikegaya Y, Koyama R. Synaptic pruning by microglia in epilepsy. J Clin Med 2019, 8: 2170. 31818018 10.3390/jcm8122170 PMC6947403

[11] Guo A, Zhang H, Li H, Chiu A, García-Rodríguez C, Lagos CF, *et al*. Inhibition of connexin hemichannels alleviates neuroinflammation and hyperexcitability in temporal lobe epilepsy. Proc Natl Acad Sci U S A 2022, 119: e2213162119. 36322757 10.1073/pnas.2213162119 PMC9659366

[12] Seifert G, Steinhäuser C. Neuron-astrocyte signaling and epilepsy. Exp Neurol 2013, 244: 4–10. 21925173 10.1016/j.expneurol.2011.08.024

[13] Weissberg I, Wood L, Kamintsky L, Vazquez O, Milikovsky DZ, Alexander A, *et al*. Albumin induces excitatory synaptogenesis through astrocytic TGF-β/ALK5 signaling in a model of acquired epilepsy following blood-brain barrier dysfunction. Neurobiol Dis 2015, 78: 115–125. 25836421 10.1016/j.nbd.2015.02.029 PMC4426044

[14] Tang F, Hartz AMS, Bauer B. Drug-resistant epilepsy: Multiple hypotheses, few answers. Front Neurol 2017, 8: 301. 28729850 10.3389/fneur.2017.00301 PMC5498483

[15] Goldsmith DR, Bekhbat M, Mehta ND, Felger JC. Inflammation-related functional and structural dysconnectivity as a pathway to psychopathology. Biol Psychiatry 2023, 93: 405–418. 36725140 10.1016/j.biopsych.2022.11.003 PMC9895884

[16] Kitzbichler MG, Aruldass AR, Barker GJ, Wood TC, Dowell NG, Hurley SA, *et al*. Peripheral inflammation is associated with micro-structural and functional connectivity changes in depression-related brain networks. Mol Psychiatry 2021, 26: 7346–7354. 34535766 10.1038/s41380-021-01272-1 PMC8872995

[17] Bu J, Yin H, Ren N, Zhu H, Xu H, Zhang R, *et al*. Structural and functional changes in the default mode network in drug-resistant epilepsy. Epilepsy Behav 2024, 151: 109593. 38157823 10.1016/j.yebeh.2023.109593

[18] Ryvlin P, Cross JH, Rheims S. Epilepsy surgery in children and adults. Lancet Neurol 2014, 13: 1114–1126. 25316018 10.1016/S1474-4422(14)70156-5

[19] Sanz-García A, Perez-Romero M, Ortega GJ. Spectral and network characterization of focal seizure types and phases. Comput Methods Programs Biomed 2022, 217: 106704. 35220198 10.1016/j.cmpb.2022.106704

[20] Wang X, Zhang G, Wang Y, Yang L, Liang Z, Cong F. One-dimensional convolutional neural networks combined with channel selection strategy for seizure prediction using long-term intracranial EEG. Int J Neural Syst 2022, 32: 2150048. 34635034 10.1142/S0129065721500489

[21] Liu Y, Razavi Hesabi Z, Cook M, Kuhlmann L. Epileptic seizure onset predicts its duration. Eur J Neurol 2022, 29: 375–381. 34725880 10.1111/ene.15166

[22] Salami P, Borzello M, Kramer MA, Westover MB, Cash SS. Quantifying seizure termination patterns reveals limited pathways to seizure end. Neurobiol Dis 2022, 165: 105645. 35104646 10.1016/j.nbd.2022.105645 PMC8860887

[23] Vezzani A, French J, Bartfai T, Baram TZ. The role of inflammation in epilepsy. Nat Rev Neurol 2011, 7: 31–40. 21135885 10.1038/nrneurol.2010.178 PMC3378051

[24] Ravizza T, Gagliardi B, Noé F, Boer K, Aronica E, Vezzani A. Innate and adaptive immunity during epileptogenesis and spontaneous seizures: Evidence from experimental models and human temporal lobe epilepsy. Neurobiol Dis 2008, 29: 142–160. 17931873 10.1016/j.nbd.2007.08.012

[25] Balosso S, Ravizza T, Pierucci M, Calcagno E, Invernizzi R, Di Giovanni G, *et al*. Molecular and functional interactions between tumor necrosis factor-alpha receptors and the glutamatergic system in the mouse hippocampus: Implications for seizure susceptibility. Neuroscience 2009, 161: 293–300. 19285115 10.1016/j.neuroscience.2009.03.005

[26] Campos-Bedolla P, Feria-Romero I, Orozco-Suárez S. Factors not considered in the study of drug-resistant epilepsy: Drug-resistant epilepsy: Assessment of neuroinflammation. Epilepsia Open 2022, 7: S68–S80. 35247028 10.1002/epi4.12590 PMC9340302

[27] Giannoni P, Badaut J, Dargazanli C, Fayd’Herbe De Maudave A, Klement W, Costalat V, *et al*. The pericyte-glia interface at the blood-brain barrier. Clin Sci 2018, 132: 361–374. 10.1042/CS20171634 29439117

[28] Klement W, Garbelli R, Zub E, Rossini L, Tassi L, Girard B, *et al*. Seizure progression and inflammatory mediators promote pericytosis and pericyte-microglia clustering at the cerebrovasculature. Neurobiol Dis 2018, 113: 70–81. 29432809 10.1016/j.nbd.2018.02.002

[29] Potschka H. Targeting regulation of ABC efflux transporters in brain diseases: A novel therapeutic approach. Pharmacol Ther 2010, 125: 118–127. 19896502 10.1016/j.pharmthera.2009.10.004

[30] Deng X, Shao Y, Xie Y, Feng Y, Wu M, Wang M, *et al*. MicroRNA-146a-5p downregulates the expression of P-glycoprotein in rats with lithium-pilocarpine-induced status epilepticus. Biol Pharm Bull 2019, 42: 744–750. 31061316 10.1248/bpb.b18-00937

[31] Varvel NH, Neher JJ, Bosch A, Wang W, Ransohoff RM, Miller RJ, *et al*. Infiltrating monocytes promote brain inflammation and exacerbate neuronal damage after status epilepticus. Proc Natl Acad Sci U S A 2016, 113: E5665–E5674. 27601660 10.1073/pnas.1604263113 PMC5035862

[32] Feng L, Murugan M, Bosco DB, Liu Y, Peng J, Worrell GA, *et al*. Microglial proliferation and monocyte infiltration contribute to microgliosis following status epilepticus. Glia 2019, 67: 1434–1448. 31179602 10.1002/glia.23616 PMC6559368

[33] Bosco DB, Tian DS, Wu LJ. Neuroimmune interaction in seizures and epilepsy: Focusing on monocyte infiltration. FEBS J 2020, 287: 4822–4837. 32473609 10.1111/febs.15428 PMC7817085

[34] Matsuo T, Komori R, Nakatani M, Ochi S, Yokota-Nakatsuma A, Matsumoto J, *et al*. Levetiracetam suppresses the infiltration of neutrophils and monocytes and downregulates many inflammatory cytokines during epileptogenesis in pilocarpine-induced status epilepticus mice. Int J Mol Sci 2022, 23: 7671. 35887020 10.3390/ijms23147671 PMC9319101

[35] Alyu F, Dikmen M. Inflammatory aspects of epileptogenesis: Contribution of molecular inflammatory mechanisms. Acta Neuropsychiatr 2017, 29: 1–16. 27692004 10.1017/neu.2016.47

[36] Librizzi L, Noè F, Vezzani A, de Curtis M, Ravizza T. Seizure-induced brain-borne inflammation sustains seizure recurrence and blood-brain barrier damage. Ann Neurol 2012, 72: 82–90. 22829270 10.1002/ana.23567

[37] Vezzani A, Balosso S, Ravizza T. Neuroinflammatory pathways as treatment targets and biomarkers in epilepsy. Nat Rev Neurol 2019, 15: 459–472. 31263255 10.1038/s41582-019-0217-x

[38] Liu X, Zhang Y, Zhao Y, Zhang Q, Han F. The neurovascular unit dysfunction in the molecular mechanisms of epileptogenesis and targeted therapy. Neurosci Bull 2024, 40: 621–634. 38564049 10.1007/s12264-024-01193-3 PMC11127907

[39] Salter MW, Stevens B. Microglia emerge as central players in brain disease. Nat Med 2017, 23: 1018–1027. 28886007 10.1038/nm.4397

[40] Herz J, Filiano AJ, Wiltbank AT, Yogev N, Kipnis J. Myeloid cells in the central nervous system. Immunity 2017, 46: 943–956. 28636961 10.1016/j.immuni.2017.06.007 PMC5657250

[41] Menassa DA, Gomez-Nicola D. Microglial dynamics during human brain development. Front Immunol 2018, 9: 1014. 29881376 10.3389/fimmu.2018.01014 PMC5976733

[42] Takahashi Y, Yu Z, Mai S, Tomita H. Linking activation of microglia and peripheral monocytic cells to the pathophysiology of psychiatric disorders. Front Cell Neurosci 2016, 10: 144. 27375431 10.3389/fncel.2016.00144 PMC4891983

[43] Eyo UB, Murugan M, Wu LJ. Microglia-neuron communication in epilepsy. Glia 2017, 65: 5–18. 27189853 10.1002/glia.23006 PMC5116010

[44] Guo S, Wang H, Yin Y. Microglia polarization from M1 to M2 in neurodegenerative diseases. Front Aging Neurosci 2022, 14: 815347. 35250543 10.3389/fnagi.2022.815347 PMC8888930

[45] Kwon HS, Koh SH. Neuroinflammation in neurodegenerative disorders: The roles of microglia and astrocytes. Transl Neurodegener 2020, 9: 42. 33239064 10.1186/s40035-020-00221-2 PMC7689983

[46] Walker DG, Lue LF. Immune phenotypes of microglia in human neurodegenerative disease: Challenges to detecting microglial polarization in human brains. Alzheimers Res Ther 2015, 7: 56. 26286145 10.1186/s13195-015-0139-9 PMC4543480

[47] Chen AQ, Fang Z, Chen XL, Yang S, Zhou YF, Mao L, *et al*. Microglia-derived TNF-α mediates endothelial necroptosis aggravating blood brain-barrier disruption after ischemic stroke. Cell Death Dis 2019, 10: 487. 31221990 10.1038/s41419-019-1716-9 PMC6586814

[48] Eyo UB, Peng J, Swiatkowski P, Mukherjee A, Bispo A, Wu LJ. Neuronal hyperactivity recruits microglial processes via neuronal NMDA receptors and microglial P2Y12 receptors after status epilepticus. J Neurosci 2014, 34: 10528–10540. 25100587 10.1523/JNEUROSCI.0416-14.2014 PMC4200107

[49] Gao C, Jiang J, Tan Y, Chen S. Microglia in neurodegenerative diseases: Mechanism and potential therapeutic targets. Signal Transduct Target Ther 2023, 8: 359. 37735487 10.1038/s41392-023-01588-0 PMC10514343

[50] Hanak TJ, Libbey JE, Doty DJ, Sim JT, DePaula-Silva AB, Fujinami RS. Positive modulation of mGluR5 attenuates seizures and reduces TNF-α^+^ macrophages and microglia in the brain in a murine model of virus-induced temporal lobe epilepsy. Exp Neurol 2019, 311: 194–204. 30316834 10.1016/j.expneurol.2018.10.006 PMC6263825

[51] Fan J, Dong X, Tang Y, Wang X, Lin D, Gong L, *et al*. Preferential pruning of inhibitory synapses by microglia contributes to alteration of the balance between excitatory and inhibitory synapses in the hippocampus in temporal lobe epilepsy. CNS Neurosci Ther 2023, 29: 2884–2900. 37072932 10.1111/cns.14224 PMC10493672

[52] Vezzani A, Fujinami RS, White HS, Preux PM, Blümcke I, Sander JW, *et al*. Infections, inflammation and epilepsy. Acta Neuropathol 2016, 131: 211–234. 26423537 10.1007/s00401-015-1481-5 PMC4867498

[53] Vezzani A, Moneta D, Richichi C, Aliprandi M, Burrows SJ, Ravizza T, *et al*. Functional role of inflammatory cytokines and antiinflammatory molecules in seizures and epileptogenesis. Epilepsia 2002, 43: 30–35. 12121291 10.1046/j.1528-1157.43.s.5.14.x

[54] Victor TR, Tsirka SE. Microglial contributions to aberrant neurogenesis and pathophysiology of epilepsy. Neuroimmunol Neuroinflamm 2020, 7: 234–247. 33154976 10.20517/2347-8659.2020.02 PMC7641338

[55] Augusto-Oliveira M, Arrifano GP, Takeda PY, Lopes-Araújo A, Santos-Sacramento L, Anthony DC, *et al*. Astroglia-specific contributions to the regulation of synapses, cognition and behaviour. Neurosci Biobehav Rev 2020, 118: 331–357. 32768488 10.1016/j.neubiorev.2020.07.039

[56] Tang Y, Zhang S, Xu C. Now we can tame the wild west of controlling astrocytes for treating neocortical epilepsy. Neurosci Bull 2023, 39: 1189–1190. 36947391 10.1007/s12264-023-01048-3 PMC10313607

[57] Escartin C, Galea E, Lakatos A, O’Callaghan JP, Petzold GC, Serrano-Pozo A, *et al*. Reactive astrocyte nomenclature, definitions, and future directions. Nat Neurosci 2021, 24: 312–325. 33589835 10.1038/s41593-020-00783-4 PMC8007081

[58] Liddelow SA, Barres BA. Reactive astrocytes: Production, function, and therapeutic potential. Immunity 2017, 46: 957–967. 28636962 10.1016/j.immuni.2017.06.006

[59] Liddelow SA, Guttenplan KA, Clarke LE, Bennett FC, Bohlen CJ, Schirmer L, *et al*. Neurotoxic reactive astrocytes are induced by activated microglia. Nature 2017, 541: 481–487. 28099414 10.1038/nature21029 PMC5404890

[60] Miyamoto N, Magami S, Inaba T, Ueno Y, Hira K, Kijima C, *et al*. The effects of A1/A2 astrocytes on oligodendrocyte linage cells against white matter injury under prolonged cerebral hypoperfusion. Glia 2020, 68: 1910–1924. 32108971 10.1002/glia.23814

[61] Liu B, Teschemacher AG, Kasparov S. Astroglia as a cellular target for neuroprotection and treatment of neuro-psychiatric disorders. Glia 2017, 65: 1205–1226. 28300322 10.1002/glia.23136 PMC5669250

[62] Xiong XY, Wang TG, Yang MH, Meng ZY, Yang QW, Wang FX. Interleukin-21 expression in hippocampal astrocytes is enhanced following kainic acid-induced seizures. Neurol Res 2016, 38: 151–157. 27118610 10.1080/01616412.2015.1135557

[63] Wang X, Yang XL, Kong WL, Zeng ML, Shao L, Jiang GT, *et al*. TRPV1 translocated to astrocytic membrane to promote migration and inflammatory infiltration thus promotes epilepsy after hypoxic ischemia in immature brain. J Neuroinflammation 2019, 16: 214. 31722723 10.1186/s12974-019-1618-x PMC6852893

[64] Wang X, Sha L, Sun N, Shen Y, Xu Q. Deletion of mTOR in reactive astrocytes suppresses chronic seizures in a mouse model of temporal lobe epilepsy. Mol Neurobiol 2017, 54: 175–187. 26732600 10.1007/s12035-015-9590-7

[65] Shlosberg D, Benifla M, Kaufer D, Friedman A. Blood-brain barrier breakdown as a therapeutic target in traumatic brain injury. Nat Rev Neurol 2010, 6: 393–403. 20551947 10.1038/nrneurol.2010.74 PMC3625732

[66] Myer DJ, Gurkoff GG, Lee SM, Hovda DA, Sofroniew MV. Essential protective roles of reactive astrocytes in traumatic brain injury. Brain 2006, 129: 2761–2772. 16825202 10.1093/brain/awl165

[67] Jha MK, Jo M, Kim JH, Suk K. Microglia-astrocyte crosstalk: An intimate molecular conversation. Neuroscientist 2019, 25: 227–240. 29931997 10.1177/1073858418783959

[68] Traiffort E, Kassoussi A, Zahaf A, Laouarem Y. Astrocytes and microglia as major players of myelin production in normal and pathological conditions. Front Cell Neurosci 2020, 14: 79. 32317939 10.3389/fncel.2020.00079 PMC7155218

[69] Lee M, Schwab C, McGeer PL. Astrocytes are GABAergic cells that modulate microglial activity. Glia 2011, 59: 152–165. 21046567 10.1002/glia.21087

[70] Xu J, Dong H, Qian Q, Zhang X, Wang Y, Jin W, *et al*. Astrocyte-derived CCL2 participates in surgery-induced cognitive dysfunction and neuroinflammation via evoking microglia activation. Behav Brain Res 2017, 332: 145–153. 28587818 10.1016/j.bbr.2017.05.066

[71] Deshpande T, Li T, Henning L, Wu Z, Müller J, Seifert G, *et al*. Constitutive deletion of astrocytic connexins aggravates kainate-induced epilepsy. Glia 2020, 68: 2136–2147. 32240558 10.1002/glia.23832

[72] Halassa MM, Fellin T, Haydon PG. The tripartite synapse: Roles for gliotransmission in health and disease. Trends Mol Med 2007, 13: 54–63. 17207662 10.1016/j.molmed.2006.12.005

[73] Perea G, Navarrete M, Araque A. Tripartite synapses: Astrocytes process and control synaptic information. Trends Neurosci 2009, 32: 421–431. 19615761 10.1016/j.tins.2009.05.001

[74] Díaz-García CM, Mongeon R, Lahmann C, Koveal D, Zucker H, Yellen G. Neuronal stimulation triggers neuronal glycolysis and not lactate uptake. Cell Metab 2017, 26: 361-374.e4. 28768175 10.1016/j.cmet.2017.06.021 PMC5559896

[75] Suzuki A, Stern SA, Bozdagi O, Huntley GW, Walker RH, Magistretti PJ, *et al*. Astrocyte-neuron lactate transport is required for long-term memory formation. Cell 2011, 144: 810–823. 21376239 10.1016/j.cell.2011.02.018 PMC3073831

[76] Huguet G, Joglekar A, Messi LM, Buckalew R, Wong S, Terman D. Neuroprotective role of gap junctions in a neuron astrocyte network model. Biophys J 2016, 111: 452–462. 27463146 10.1016/j.bpj.2016.05.051 PMC4968398

[77] de Curtis M, Garbelli R, Uva L. A hypothesis for the role of axon demyelination in seizure generation. Epilepsia 2021, 62: 583–595. 33493363 10.1111/epi.16824

[78] Vo AH, Ambady P, Spencer D. The IDH1 inhibitor ivosidenib improved seizures in a patient with drug-resistant epilepsy from IDH1 mutant oligodendroglioma. Epilepsy Behav Rep 2022, 18: 100526. 35198955 10.1016/j.ebr.2022.100526 PMC8844211

[79] Kumar P, Lim A, Hazirah SN, Chua CJH, Ngoh A, Poh SL, *et al*. Single-cell transcriptomics and surface epitope detection in human brain epileptic lesions identifies pro-inflammatory signaling. Nat Neurosci 2022, 25: 956–966. 35739273 10.1038/s41593-022-01095-5 PMC9276529

[80] Lapato AS, Szu JI, Hasselmann JPC, Khalaj AJ, Binder DK, Tiwari-Woodruff SK. Chronic demyelination-induced seizures. Neuroscience 2017, 346: 409–422. 28153692 10.1016/j.neuroscience.2017.01.035 PMC5394933

[81] Hoffmann K, Lindner M, Gröticke I, Stangel M, Löscher W. Epileptic seizures and hippocampal damage after cuprizone-induced demyelination in C57BL/6 mice. Exp Neurol 2008, 210: 308–321. 18096162 10.1016/j.expneurol.2007.11.005

[82] Wilkins A, Majed H, Layfield R, Compston A, Chandran S. Oligodendrocytes promote neuronal survival and axonal length by distinct intracellular mechanisms: A novel role for oligodendrocyte-derived glial cell line-derived neurotrophic factor. J Neurosci 2003, 23: 4967–4974. 12832519 10.1523/JNEUROSCI.23-12-04967.2003 PMC6741206

[83] Ubhi K, Rockenstein E, Mante M, Inglis C, Adame A, Patrick C, *et al*. Neurodegeneration in a transgenic mouse model of multiple system atrophy is associated with altered expression of oligodendroglial-derived neurotrophic factors. J Neurosci 2010, 30: 6236–6246. 20445049 10.1523/JNEUROSCI.0567-10.2010 PMC2896284

[84] Aronica E, Crino PB. Inflammation in epilepsy: Clinical observations. Epilepsia 2011, 52: 26–32. 21542843 10.1111/j.1528-1167.2011.03033.x

[85] Vieira ÉLM, de Oliveira GNM, Lessa JMK, Gonçalves AP, Oliveira ACP, Bauer ME, *et al*. Peripheral leukocyte profile in people with temporal lobe epilepsy reflects the associated proinflammatory state. Brain Behav Immun 2016, 53: 123–130. 26640228 10.1016/j.bbi.2015.11.016

[86] Toledo A, Orozco-Suárez S, Rosetti M, Maldonado L, Bautista SI, Flores X, *et al*. Temporal lobe epilepsy: Evaluation of central and systemic immune-inflammatory features associated with drug resistance. Seizure 2021, 91: 447–455. 34340190 10.1016/j.seizure.2021.07.028

[87] Xu D, Robinson AP, Ishii T, Duncan DS, Alden TD, Goings GE, *et al*. Peripherally derived T regulatory and γδ T cells have opposing roles in the pathogenesis of intractable pediatric epilepsy. J Exp Med 2018, 215: 1169–1186. 29487082 10.1084/jem.20171285 PMC5881465

[88] Ni FF, Li CR, Liao JX, Wang GB, Lin SF, Xia Y, *et al*. The effects of ketogenic diet on the Th17/Treg cells imbalance in patients with intractable childhood epilepsy. Seizure 2016, 38: 17–22. 27061881 10.1016/j.seizure.2016.03.006

[89] Spatola M, Dalmau J. Seizures and risk of epilepsy in autoimmune and other inflammatory encephalitis. Curr Opin Neurol 2017, 30: 345–353. 28234800 10.1097/WCO.0000000000000449 PMC5831325

[90] Wesselingh R, Broadley J, Buzzard K, Tarlinton D, Seneviratne U, Kyndt C, *et al*. Prevalence, risk factors, and prognosis of drug-resistant epilepsy in autoimmune encephalitis. Epilepsy Behav 2022, 132: 108729. 35623203 10.1016/j.yebeh.2022.108729

[91] Flammer J, Neziraj T, Rüegg S, Pröbstel AK. Immune mechanisms in epileptogenesis: Update on diagnosis and treatment of autoimmune epilepsy syndromes. Drugs 2023, 83: 135–158. 36696027 10.1007/s40265-022-01826-9 PMC9875200

[92] Kurukumbi M, Dave RH, Castillo J, Shah T, Lau J. Rituximab for autoimmune encephalitis with epilepsy. Case Rep Neurol Med 2020, 2020: 5843089. 32655958 10.1155/2020/5843089 PMC7330651

[93] Hachiya Y, Uruha A, Kasai-Yoshida E, Shimoda K, Satoh-Shirai I, Kumada S, *et al*. Rituximab ameliorates anti-N-methyl-D-aspartate receptor encephalitis by removal of short-lived plasmablasts. J Neuroimmunol 2013, 265: 128–130. 24183642 10.1016/j.jneuroim.2013.09.017

[94] Zimmer TS, Broekaart DWM, Luinenburg M, Mijnsbergen C, Anink JJ, Sim NS, *et al*. Balloon cells promote immune system activation in focal cortical dysplasia type 2b. Neuropathol Appl Neurobiol 2021, 47: 826–839. 34003514 10.1111/nan.12736 PMC8518746

[95] Zattoni M, Mura ML, Deprez F, Schwendener RA, Engelhardt B, Frei K, *et al*. Brain infiltration of leukocytes contributes to the pathophysiology of temporal lobe epilepsy. J Neurosci 2011, 31: 4037–4050. 21411646 10.1523/JNEUROSCI.6210-10.2011 PMC6623535

[96] Penkowa M, Quintana A, Carrasco J, Giralt M, Molinero A, Hidalgo J. Metallothionein prevents neurodegeneration and central nervous system cell death after treatment with gliotoxin 6-aminonicotinamide. J Neurosci Res 2004, 77: 35–53. 15197737 10.1002/jnr.20154

[97] Gerard C, Rollins BJ. Chemokines and disease. Nat Immunol 2001, 2: 108–115. 11175802 10.1038/84209

[98] Krumbholz M, Theil D, Cepok S, Hemmer B, Kivisäkk P, Ransohoff RM, *et al*. Chemokines in multiple sclerosis: CXCL12 and CXCL13 up-regulation is differentially linked to CNS immune cell recruitment. Brain 2006, 129: 200–211. 16280350 10.1093/brain/awh680

[99] Larochelle C, Wasser B, Jamann H, Löffel JT, Cui QL, Tastet O, *et al*. Pro-inflammatory T helper 17 directly harms oligodendrocytes in neuroinflammation. Proc Natl Acad Sci U S A 2021, 118: e2025813118. 34417310 10.1073/pnas.2025813118 PMC8403833

[100] Kebir H, Kreymborg K, Ifergan I, Dodelet-Devillers A, Cayrol R, Bernard M, *et al*. Human TH17 lymphocytes promote blood-brain barrier disruption and central nervous system inflammation. Nat Med 2007, 13: 1173–1175. 17828272 10.1038/nm1651 PMC5114125

[101] Lu L, Pan K, Zheng HX, Li JJ, Qiu HJ, Zhao JJ, *et al*. IL-17A promotes immune cell recruitment in human esophageal cancers and the infiltrating dendritic cells represent a positive prognostic marker for patient survival. J Immunother 2013, 36: 451–458. 23994890 10.1097/CJI.0b013e3182a802cf

[102] Chen Y, Nagib MM, Yasmen N, Sluter MN, Littlejohn TL, Yu Y, *et al*. Neuroinflammatory mediators in acquired epilepsy: An update. Inflamm Res 2023, 72: 683–701. 36745211 10.1007/s00011-023-01700-8 PMC10262518

[103] Cai M, Lin W. The function of NF-kappa B during epilepsy, a potential therapeutic target. Front Neurosci 2022, 16: 851394. 35360161 10.3389/fnins.2022.851394 PMC8961383

[104] Tang X, Chen X, Li X, Cheng H, Gan J, Liu Z. The TLR4 mediated inflammatory signal pathway might be involved in drug resistance in drug-resistant epileptic rats. J Neuroimmunol 2022, 365: 577802. 35217365 10.1016/j.jneuroim.2021.577802

[105] Henneberger C, Steinhäuser C. Astrocytic TLR4 at the crossroads of inflammation and seizure susceptibility. J Cell Biol 2016, 215: 607–609. 27881712 10.1083/jcb.201611078 PMC5147008

[106] Lagarde S, Villeneuve N, Trébuchon A, Kaphan E, Lepine A, McGonigal A, *et al*. Anti-tumor necrosis factor alpha therapy (adalimumab) in Rasmussen’s encephalitis: An open pilot study. Epilepsia 2016, 57: 956–966. 27106864 10.1111/epi.13387

[107] Coll RC, Robertson AAB, Chae JJ, Higgins SC, Muñoz-Planillo R, Inserra MC, *et al*. A small-molecule inhibitor of the NLRP3 inflammasome for the treatment of inflammatory diseases. Nat Med 2015, 21: 248–255. 25686105 10.1038/nm.3806 PMC4392179

[108] Zubareva OE, Sinyak DS, Kalita AD, Griflyuk AV, Diespirov GP, Postnikova TY, *et al*. Antiepileptogenic effects of anakinra, lamotrigine and their combination in a lithium-pilocarpine model of temporal lobe epilepsy in rats. Int J Mol Sci 2023, 24: 15400. 37895080 10.3390/ijms242015400 PMC10607594

[109] Leitner K, Al Shammary M, McLane M, Johnston MV, Elovitz MA, Burd I. IL-1 receptor blockade prevents fetal cortical brain injury but not preterm birth in a mouse model of inflammation-induced preterm birth and perinatal brain injury. Am J Reprod Immunol 2014, 71: 418–426. 24592965 10.1111/aji.12216 PMC3989434

[110] Yamanaka G, Ishida Y, Kanou K, Suzuki S, Watanabe Y, Takamatsu T, *et al*. Towards a treatment for neuroinflammation in epilepsy: Interleukin-1 receptor antagonist, anakinra, as a potential treatment in intractable epilepsy. Int J Mol Sci 2021, 22: 6282. 34208064 10.3390/ijms22126282 PMC8230637

[111] Mohseni-Moghaddam P, Roghani M, Khaleghzadeh-Ahangar H, Sadr SS, Sala C. A literature overview on epilepsy and inflammasome activation. Brain Res Bull 2021, 172: 229–235. 33964347 10.1016/j.brainresbull.2021.05.001

[112] Xu C, Zhang S, Gong Y, Nao J, Shen Y, Tan B, *et al*. Subicular caspase-1 contributes to pharmacoresistance in temporal lobe epilepsy. Ann Neurol 2021, 90: 377–390. 34288031 10.1002/ana.26173

[113] Sitovskaya D, Zabrodskaya Y, Parshakov P, Sokolova T, Kudlay D, Starshinova A, *et al*. Expression of cytoskeletal proteins (GFAP, vimentin), proapoptotic protein (caspase-3) and protective protein (S100) in the epileptic focus in adults and children with drug-resistant temporal lobe epilepsy associated with focal cortical dysplasia. Int J Mol Sci 2023, 24: 14490. 37833937 10.3390/ijms241914490 PMC10572279

[114] Bien CG, Granata T, Antozzi C, Cross JH, Dulac O, Kurthen M, *et al*. Pathogenesis, diagnosis and treatment of Rasmussen encephalitis: A European consensus statement. Brain 2005, 128: 454–471. 15689357 10.1093/brain/awh415

[115] Bien CG, Bauer J, Deckwerth TL, Wiendl H, Deckert M, Wiestler OD, *et al*. Destruction of neurons by cytotoxic T cells: A new pathogenic mechanism in Rasmussen’s encephalitis. Ann Neurol 2002, 51: 311–318. 11891826 10.1002/ana.10100

[116] Bauer J, Elger CE, Hans VH, Schramm J, Urbach H, Lassmann H, *et al*. Astrocytes are a specific immunological target in Rasmussen’s encephalitis. Ann Neurol 2007, 62: 67–80. 17503512 10.1002/ana.21148

[117] Varadkar S, Bien CG, Kruse CA, Jensen FE, Bauer J, Pardo CA, *et al*. Rasmussen’s encephalitis: Clinical features, pathobiology, and treatment advances. Lancet Neurol 2014, 13: 195–205. 24457189 10.1016/S1474-4422(13)70260-6 PMC4005780

[118] Bien CG, Widman G, Urbach H, Sassen R, Kuczaty S, Wiestler OD, *et al*. The natural history of Rasmussen’s encephalitis. Brain 2002, 125: 1751–1759. 12135966 10.1093/brain/awf176

[119] Kebir H, Carmant L, Fontaine F, Béland K, Bosoi CM, Sanon NT, *et al*. Humanized mouse model of Rasmussen’s encephalitis supports the immune-mediated hypothesis. J Clin Invest 2018, 128: 2000–2009. 29629902 10.1172/JCI97098 PMC5919802

[120] Wang N, Mi X, Gao B, Gu J, Wang W, Zhang Y, *et al*. Minocycline inhibits brain inflammation and attenuates spontaneous recurrent seizures following pilocarpine-induced status epilepticus. Neuroscience 2015, 287: 144–156. 25541249 10.1016/j.neuroscience.2014.12.021

[121] DeSena AD, Do T, Schulert GS. Systemic autoinflammation with intractable epilepsy managed with interleukin-1 blockade. J Neuroinflammation 2018, 15: 38. 29426321 10.1186/s12974-018-1063-2 PMC5807745

[122] Leng F, Edison P. Neuroinflammation and microglial activation in Alzheimer disease: Where do we go from here? Nat Rev Neurol 2021, 17: 157–172. 33318676 10.1038/s41582-020-00435-y

[123] Hirsch EC, Hunot S. Neuroinflammation in Parkinson’s disease: A target for neuroprotection? Lancet Neurol 2009, 8: 382–397. 19296921 10.1016/S1474-4422(09)70062-6

[124] Iadecola C, Anrather J. Stroke research at a crossroad: Asking the brain for directions. Nat Neurosci 2011, 14: 1363–1368. 22030546 10.1038/nn.2953 PMC3633153

[125] Alsbrook DL, Di Napoli M, Bhatia K, Biller J, Andalib S, Hinduja A, *et al*. Neuroinflammation in acute ischemic and hemorrhagic stroke. Curr Neurol Neurosci Rep 2023, 23: 407–431. 37395873 10.1007/s11910-023-01282-2 PMC10544736

[126] Hambardzumyan D, Gutmann DH, Kettenmann H. The role of microglia and macrophages in glioma maintenance and progression. Nat Neurosci 2016, 19: 20–27. 26713745 10.1038/nn.4185 PMC4876023

[127] Gleichgerrcht E, Kocher M, Bonilha L. Connectomics and graph theory analyses: Novel insights into network abnormalities in epilepsy. Epilepsia 2015, 56: 1660–1668. 26391203 10.1111/epi.13133

[128] Amorim-Leite R, Remick M, Welch W, Abel TJ. History of the network approach in epilepsy surgery. Neurosurg Clin N Am 2020, 31: 301–308. 32475480 10.1016/j.nec.2020.03.011

[129] Johnson GW, Doss DJ, Englot DJ. Network dysfunction in pre and postsurgical epilepsy: Connectomics as a tool and not a destination. Curr Opin Neurol 2022, 35: 196–201. 34799514 10.1097/WCO.0000000000001008 PMC8891078

[130] Watrin F, Manent JB, Cardoso C, Represa A. Causes and consequences of gray matter heterotopia. CNS Neurosci Ther 2015, 21: 112–122. 25180909 10.1111/cns.12322 PMC6495304

[131] Ashraf-Ganjouei A, Rahmani F, Aarabi MH, Sanjari Moghaddam H, Nazem-Zadeh MR, Davoodi-Bojd E, *et al*. White matter correlates of disease duration in patients with temporal lobe epilepsy: Updated review of literature. Neurol Sci 2019, 40: 1209–1216. 30868482 10.1007/s10072-019-03818-2

[132] Voets NL, Bernhardt BC, Kim H, Yoon U, Bernasconi N. Increased temporolimbic cortical folding complexity in temporal lobe epilepsy. Neurology 2011, 76: 138–144. 21148116 10.1212/WNL.0b013e318205d521 PMC3030232

[133] Hatton SN, Huynh KH, Bonilha L, Abela E, Alhusaini S, Altmann A, *et al*. White matter abnormalities across different epilepsy syndromes in adults: An *ENIGMA*-Epilepsy study. Brain 2020, 143: 2454–2473. 32814957 10.1093/brain/awaa200 PMC7567169

[134] Zweiphenning WJEM, von Ellenrieder N, Dubeau F, Martineau L, Minotti L, Hall JA, *et al*. Correcting for physiological ripples improves epileptic focus identification and outcome prediction. Epilepsia 2022, 63: 483–496. 34919741 10.1111/epi.17145 PMC9300035

[135] Frauscher B, Bartolomei F, Kobayashi K, Cimbalnik J, van 't Klooster MA, Rampp S, *et al*. High-frequency oscillations: The state of clinical research. Epilepsia 2017, 58: 1316–1329. 10.1111/epi.13829 PMC5806699 28666056

[136] Cauda F, Nani A, Manuello J, Premi E, Palermo S, Tatu K, *et al*. Brain structural alterations are distributed following functional, anatomic and genetic connectivity. Brain 2018, 141: 3211–3232. 30346490 10.1093/brain/awy252 PMC6202577

[137] Lagarde S, Roehri N, Lambert I, Trebuchon A, McGonigal A, Carron R, *et al*. Interictal stereotactic-EEG functional connectivity in refractory focal epilepsies. Brain 2018, 141: 2966–2980. 30107499 10.1093/brain/awy214

[138] Shih JJ. It’s all about the networks. Epilepsy Curr 2019, 19: 165–167. 31032667 10.1177/1535759719843301 PMC6610390

[139] Williams JA, Burgess S, Suckling J, Lalousis PA, Batool F, Griffiths SL, *et al*. Inflammation and brain structure in schizophrenia and other neuropsychiatric disorders: A Mendelian randomization study. JAMA Psychiatry 2022, 79: 498–507. 35353173 10.1001/jamapsychiatry.2022.0407 PMC8968718

[140] Powell F, Tosun D, Sadeghi R, Weiner M, Raj A, Initiative ADN. Preserved structural network organization mediates pathology spread in Alzheimer’s disease spectrum despite loss of white matter tract integrity. J Alzheimers Dis 2018, 65: 747–764. 29578480 10.3233/JAD-170798 PMC6152926

[141] Lee HW, Arora J, Papademetris X, Tokoglu F, Negishi M, Scheinost D, *et al*. Altered functional connectivity in seizure onset zones revealed by fMRI intrinsic connectivity. Neurology 2014, 83: 2269–2277. 25391304 10.1212/WNL.0000000000001068 PMC4277677

[142] Bernasconi A, Cendes F, Theodore WH, Gill RS, Koepp MJ, Hogan RE, *et al*. Recommendations for the use of structural magnetic resonance imaging in the care of patients with epilepsy: A consensus report from the International League Against Epilepsy Neuroimaging Task Force. Epilepsia 2019, 60: 1054–1068. 31135062 10.1111/epi.15612

[143] Liu M, Chen Z, Beaulieu C, Gross DW. Disrupted anatomic white matter network in left mesial temporal lobe epilepsy. Epilepsia 2014, 55: 674–682. 24650167 10.1111/epi.12581

[144] Iliff JJ, Wang M, Liao Y, Plogg BA, Peng W, Gundersen GA, *et al*. A paravascular pathway facilitates CSF flow through the brain parenchyma and the clearance of interstitial solutes, including amyloid Β. Sci Transl Med 2012, 4: 147ra111. 10.1126/scitranslmed.3003748 PMC3551275 22896675

[145] Formolo DA, Yu J, Lin K, Tsang HWH, Ou H, Kranz GS, *et al*. Leveraging the glymphatic and meningeal lymphatic systems as therapeutic strategies in Alzheimer’s disease: An updated overview of nonpharmacological therapies. Mol Neurodegener 2023, 18: 26. 37081555 10.1186/s13024-023-00618-3 PMC10116684

[146] Goulay R, Flament J, Gauberti M, Naveau M, Pasquet N, Gakuba C, *et al*. Subarachnoid hemorrhage severely impairs brain parenchymal cerebrospinal fluid circulation in nonhuman primate. Stroke 2017, 48: 2301–2305. 28526764 10.1161/STROKEAHA.117.017014

[147] Peters ME, Lyketsos CG. The glymphatic system’s role in traumatic brain injury-related neurodegeneration. Mol Psychiatry 2023, 28: 2707–2715. 37185960 10.1038/s41380-023-02070-7

[148] Kiviniemi V, Wang X, Korhonen V, Keinänen T, Tuovinen T, Autio J, *et al*. Ultra-fast magnetic resonance encephalography of physiological brain activity - Glymphatic pulsation mechanisms? J Cereb Blood Flow Metab 2016, 36: 1033–1045. 26690495 10.1177/0271678X15622047 PMC4908626

[149] Andica C, Kamagata K, Takabayashi K, Kikuta J, Kaga H, Someya Y, *et al*. Neuroimaging findings related to glymphatic system alterations in older adults with metabolic syndrome. Neurobiol Dis 2023, 177: 105990. 36621631 10.1016/j.nbd.2023.105990

[150] Birg A, van der Horn HJ, Ryman SG, Branzoli F, Deelchand DK, Quinn DK, *et al*. Diffusion magnetic resonance spectroscopy captures microglial reactivity related to gut-derived systemic lipopolysaccharide: A preliminary study. Brain Behav Immun 2024, 122: 345–352. 39163909 10.1016/j.bbi.2024.08.034 PMC11418836

[151] Tavakol S, Royer J, Lowe AJ, Bonilha L, Tracy JI, Jackson GD, *et al*. Neuroimaging and connectomics of drug-resistant epilepsy at multiple scales: From focal lesions to macroscale networks. Epilepsia 2019, 60: 593–604. 30889276 10.1111/epi.14688 PMC6447443

[152] Wang KL, Hu W, Liu TH, Zhao XB, Han CL, Xia XT, *et al*. Metabolic covariance networks combining graph theory measuring aberrant topological patterns in mesial temporal lobe epilepsy. CNS Neurosci Ther 2019, 25: 396–408. 30298594 10.1111/cns.13073 PMC6488969

[153] Vivash L, O’Brien TJ. Imaging microglial activation with TSPO PET: Lighting up neurologic diseases? J Nucl Med 2016, 57: 165–168. 26697963 10.2967/jnumed.114.141713

[154] Sequeira KM, Tabesh A, Sainju RK, DeSantis SM, Naselaris T, Joseph JE, *et al*. Perfusion network shift during seizures in medial temporal lobe epilepsy. PLoS One 2013, 8: e53204. 23341932 10.1371/journal.pone.0053204 PMC3544909

[155] Zhang L, Hu K, Shao T, Hou L, Zhang S, Ye W, *et al*. Recent developments on PET radiotracers for TSPO and their applications in neuroimaging. Acta Pharm Sin B 2021, 11: 373–393. 33643818 10.1016/j.apsb.2020.08.006 PMC7893127

[156] Gershen LD, Zanotti-Fregonara P, Dustin IH, Liow JS, Hirvonen J, Kreisl WC, *et al*. Neuroinflammation in temporal lobe epilepsy measured using positron emission tomographic imaging of translocator protein. JAMA Neurol 2015, 72: 882–888. 26052981 10.1001/jamaneurol.2015.0941 PMC5234042

[157] Bouilleret V, Dedeurwaerdere S. What value can TSPO PET bring for epilepsy treatment? Eur J Nucl Med Mol Imaging 2021, 49: 221–233. 34120191 10.1007/s00259-021-05449-2

[158] Chugani DC. α-methyl-L-tryptophan: Mechanisms for tracer localization of epileptogenic brain regions. Biomark Med 2011, 5: 567–575. 22003905 10.2217/bmm.11.73 PMC3399668

[159] Stępień KM, Tomaszewski M, Tomaszewska J, Czuczwar SJ. The multidrug transporter P-glycoprotein in pharmacoresistance to antiepileptic drugs. Pharmacol Rep 2012, 64: 1011–1019. 23238460 10.1016/s1734-1140(12)70900-3

[160] Ballweg A, Klaus C, Vogler L, Katzdobler S, Wind K, Zatcepin A, *et al*. ^18^F]F-DED PET imaging of reactive astrogliosis in neurodegenerative diseases: Preclinical proof of concept and first-in-human data. J Neuroinflammation 2023, 20: 68. 36906584 10.1186/s12974-023-02749-2 PMC10007845

[161] Bagic A, Funke ME, Ebersole J, Position Statement Committee ACMESS. American Clinical MEG Society (ACMEGS) position statement: The value of magnetoencephalography (MEG)/magnetic source imaging (MSI) in noninvasive presurgical evaluation of patients with medically intractable localization-related epilepsy. J Clin Neurophysiol 2009, 26: 290–293. 10.1097/WNP.0b013e3181b49d50 19602984

[162] Mégevand P, Seeck M. Electroencephalography, magnetoencephalography and source localization: Their value in epilepsy. Curr Opin Neurol 2018, 31: 176–183. 29432218 10.1097/WCO.0000000000000545

[163] Ye H, Chen C, Weiss SA, Wang S. Pathological and physiological high-frequency oscillations on electroencephalography in patients with epilepsy. Neurosci Bull 2024, 40: 609–620. 37999861 10.1007/s12264-023-01150-6 PMC11127900

[164] Cao M, Galvis D, Vogrin SJ, Woods WP, Vogrin S, Wang F, *et al*. Virtual intracranial EEG signals reconstructed from MEG with potential for epilepsy surgery. Nat Commun 2022, 13: 994. 35194035 10.1038/s41467-022-28640-x PMC8863890

[165] Cao M, Vogrin SJ, Peterson ADH, Woods W, Cook MJ, Plummer C. Dynamical network models from EEG and MEG for epilepsy surgery-a quantitative approach. Front Neurol 2022, 13: 837893. 35422755 10.3389/fneur.2022.837893 PMC9001937

[166] Staljanssens W, Strobbe G, Van Holen R, Keereman V, Gadeyne S, Carrette E, *et al*. EEG source connectivity to localize the seizure onset zone in patients with drug resistant epilepsy. Neuroimage Clin 2017, 16: 689–698. 29034162 10.1016/j.nicl.2017.09.011 PMC5633847

[167] Singh J, Ebersole JS, Brinkmann BH. From theory to practical fundamentals of electroencephalographic source imaging in localizing the epileptogenic zone. Epilepsia 2022, 63: 2476–2490. 35811476 10.1111/epi.17361 PMC9796417

[168] Saez I, Gu X. Invasive computational psychiatry. Biol Psychiatry 2023, 93: 661–670. 36641365 10.1016/j.biopsych.2022.09.032 PMC10038930

[169] Zhang H, Zhou QQ, Chen H, Hu XQ, Li WG, Bai Y, *et al*. The applied principles of EEG analysis methods in neuroscience and clinical neurology. Mil Med Res 2023, 10: 67. 38115158 10.1186/s40779-023-00502-7 PMC10729551

[170] Slinger G, Otte WM, Braun KPJ, van Diessen E. An updated systematic review and meta-analysis of brain network organization in focal epilepsy: Looking back and forth. Neurosci Biobehav Rev 2022, 132: 211–223. 34813826 10.1016/j.neubiorev.2021.11.028

[171] Meisel C, Bailey KA. Identifying signal-dependent information about the preictal state: A comparison across ECoG, EEG and EKG using deep learning. EBioMedicine 2019, 45: 422–431. 31300348 10.1016/j.ebiom.2019.07.001 PMC6642360

[172] Wang HE, Woodman M, Triebkorn P, Lemarechal JD, Jha J, Dollomaja B, *et al*. Delineating epileptogenic networks using brain imaging data and personalized modeling in drug-resistant epilepsy. Sci Transl Med 2023, 15: eabp8982. 10.1126/scitranslmed.abp8982 36696482

[173] Borbély K, Emri M, Kenessey I, Tóth M, Singer J, Barsi P, *et al*. PET/MRI in the presurgical evaluation of patients with epilepsy: A concordance analysis. Biomedicines 2022, 10: 949. 35625684 10.3390/biomedicines10050949 PMC9138772

[174] Guo K, Cui B, Shang K, Hou Y, Fan X, Yang H, *et al*. Assessment of localization accuracy and postsurgical prediction of simultaneous ^18^F-FDG PET/MRI in refractory epilepsy patients. Eur Radiol 2021, 31: 6974–6982. 33638688 10.1007/s00330-021-07738-8

[175] Szaflarski JP, DiFrancesco M, Hirschauer T, Banks C, Privitera MD, Gotman J, *et al*. Cortical and subcortical contributions to absence seizure onset examined with EEG/fMRI. Epilepsy Behav 2010, 18: 404–413. 20580319 10.1016/j.yebeh.2010.05.009 PMC2922486

[176] Murakami H, Wang ZI, Marashly A, Krishnan B, Prayson RA, Kakisaka Y, *et al*. Correlating magnetoencephalography to stereo-electroencephalography in patients undergoing epilepsy surgery. Brain 2016, 139: 2935–2947. 27567464 10.1093/brain/aww215 PMC5091043

[177] Krishnan B, Tousseyn S, Wang ZI, Murakami H, Wu G, Burgess R, *et al*. Novel noninvasive identification of patient-specific epileptic networks in focal epilepsies: Linking single-photon emission computed tomography perfusion during seizures with resting-state magnetoencephalography dynamics. Hum Brain Mapp 2023, 44: 1695–1710. 36480260 10.1002/hbm.26168 PMC9921232

[178] Larivière S, Royer J, Rodríguez-Cruces R, Paquola C, Caligiuri ME, Gambardella A, *et al*. Structural network alterations in focal and generalized epilepsy assessed in a worldwide *ENIGMA* study follow axes of epilepsy risk gene expression. Nat Commun 2022, 13: 4320. 35896547 10.1038/s41467-022-31730-5 PMC9329287

[179] Bonilha L, Nesland T, Martz GU, Joseph JE, Spampinato MV, Edwards JC, *et al*. Medial temporal lobe epilepsy is associated with neuronal fibre loss and paradoxical increase in structural connectivity of limbic structures. J Neurol Neurosurg Psychiatry 2012, 83: 903–909. 22764263 10.1136/jnnp-2012-302476 PMC3415309

[180] Amiri S, Mehvari-Habibabadi J, Mohammadi-Mobarakeh N, Hashemi-Fesharaki SS, Mirbagheri MM, Elisevich K, *et al*. Graph theory application with functional connectivity to distinguish left from right temporal lobe epilepsy. Epilepsy Res 2020, 167: 106449. 32937221 10.1016/j.eplepsyres.2020.106449

[181] Zhang Z, Liao W, Xu Q, Wei W, Zhou HJ, Sun K, *et al*. Hippocampus-associated causal network of structural covariance measuring structural damage progression in temporal lobe epilepsy. Hum Brain Mapp 2017, 38: 753–766. 27677885 10.1002/hbm.23415 PMC6866709

[182] Pizzanelli C, Pesaresi I, Milano C, Cecchi P, Fontanelli L, Giannoni S, *et al*. Distinct limbic connectivity in left and right benign mesial temporal lobe epilepsy: Evidence from a resting state functional MRI study. Front Neurol 2022, 13: 943660. 36247782 10.3389/fneur.2022.943660 PMC9558280

[183] Guedj E, Bonini F, Gavaret M, Trébuchon A, Aubert S, Boucekine M, *et al*. 18FDG-PET in different subtypes of temporal lobe epilepsy: SEEG validation and predictive value. Epilepsia 2015, 56: 414–421. 25708545 10.1111/epi.12917

[184] Yasuda CL, Chen Z, Beltramini GC, Coan AC, Morita ME, Kubota B, *et al*. Aberrant topological patterns of brain structural network in temporal lobe epilepsy. Epilepsia 2015, 56: 1992–2002. 26530395 10.1111/epi.13225

[185] Vanicek T, Hahn A, Traub-Weidinger T, Hilger E, Spies M, Wadsak W, *et al*. Insights into Intrinsic Brain Networks based on Graph Theory and PET in right- compared to left-sided Temporal Lobe Epilepsy. Sci Rep 2016, 6: 28513. 27349503 10.1038/srep28513 PMC4923886

[186] Liu C, Zhao XM, Wang Q, Du TT, Zhang MX, Wang HZ, *et al*. Astrocyte-derived SerpinA3N promotes neuroinflammation and epileptic seizures by activating the NF-κB signaling pathway in mice with temporal lobe epilepsy. J Neuroinflammation 2023, 20: 161. 37422673 10.1186/s12974-023-02840-8 PMC10329806

[187] Liang XS, Qian TL, Xiong YF, Liang XT, Ding YW, Zhu XY, *et al*. IRAK-M ablation promotes status epilepticus-induced neuroinflammation via activating M1 microglia and impairing excitatory synaptic function. Mol Neurobiol 2023, 60: 5199–5213. 37277682 10.1007/s12035-023-03407-7

[188] Bryant L, McKinnon ET, Taylor JA, Jensen JH, Bonilha L, de Bezenac C, *et al*. Fiber ball white matter modeling in focal epilepsy. Hum Brain Mapp 2021, 42: 2490–2507. 33605514 10.1002/hbm.25382 PMC8090772

[189] Pierre WC, Zhang E, Londono I, De Leener B, Lesage F, Lodygensky GA. Non-invasive *in vivo* MRI detects long-term microstructural brain alterations related to learning and memory impairments in a model of inflammation-induced white matter injury. Behav Brain Res 2022, 428: 113884. 35398230 10.1016/j.bbr.2022.113884

[190] Frigerio F, Pasqualini G, Craparotta I, Marchini S, van Vliet EA, Foerch P, *et al*. N-3 Docosapentaenoic acid-derived protectin D1 promotes resolution of neuroinflammation and arrests epileptogenesis. Brain 2018, 141: 3130–3143. 30307467 10.1093/brain/awy247 PMC6202571

[191] Lajkó N, Kata, Szabó M, Mátyás A, Dulka K, Földesi I, *et al*. Sensitivity of rodent microglia to kynurenines in models of epilepsy and inflammation *in vivo* and in vitro: Microglia activation is inhibited by kynurenic acid and the synthetic analogue SZR104. Int J Mol Sci 2020, 21: 9333. 10.3390/ijms21239333 PMC7731372 33297593

[192] Fan Y, Wang W, Li W, Li X. MiR-15a inhibits cell apoptosis and inflammation in a temporal lobe epilepsy model by downregulating GFAP. Mol Med Rep 2020, 22: 3504–3512. 32945401 10.3892/mmr.2020.11388

[193] Pauletti A, Terrone G, Shekh-Ahmad T, Salamone A, Ravizza T, Rizzi M, *et al*. Targeting oxidative stress improves disease outcomes in a rat model of acquired epilepsy. Brain 2019, 142: e39. 31145451 10.1093/brain/awz130 PMC6598637

[194] Devinsky O, Vezzani A, Najjar S, De Lanerolle NC, Rogawski MA. *Glia* and epilepsy: Excitability and inflammation. Trends Neurosci 2013, 36: 174–184. 23298414 10.1016/j.tins.2012.11.008

[195] Madireddy S, Madireddy S. Therapeutic strategies to ameliorate neuronal damage in epilepsy by regulating oxidative stress, mitochondrial dysfunction, and neuroinflammation. Brain Sci 2023, 13: 784. 37239256 10.3390/brainsci13050784 PMC10216584

[196] Toi PT, Jang HJ, Min K, Kim SP, Lee SK, Lee J, *et al*. *In vivo* direct imaging of neuronal activity at high temporospatial resolution. Science 2022, 378: 160–168. 36227975 10.1126/science.abh4340

[197] Gong Q, He Y. Depression, neuroimaging and connectomics: A selective overview. Biol Psychiatry 2015, 77: 223–235. 25444171 10.1016/j.biopsych.2014.08.009

[198] Liu M, Bernhardt BC, Hong SJ, Caldairou B, Bernasconi A, Bernasconi N. The superficial white matter in temporal lobe epilepsy: A key link between structural and functional network disruptions. Brain 2016, 139: 2431–2440. 27357350 10.1093/brain/aww167 PMC4995361

[199] Li X, Jiang Y, Li W, Qin Y, Li Z, Chen Y, *et al*. Disrupted functional connectivity in white matter resting-state networks in unilateral temporal lobe epilepsy. Brain Imaging Behav 2022, 16: 324–335. 34478055 10.1007/s11682-021-00506-8

[200] Bartolomei F, Lagarde S, Wendling F, McGonigal A, Jirsa V, Guye M, *et al*. Defining epileptogenic networks: Contribution of SEEG and signal analysis. Epilepsia 2017, 58: 1131–1147. 28543030 10.1111/epi.13791

[201] Song J, Nair VA, Gaggl W, Prabhakaran V. Disrupted brain functional organization in epilepsy revealed by graph theory analysis. Brain Connect 2015, 5: 276–283. 25647011 10.1089/brain.2014.0308 PMC4490776

[202] Výtvarová E, Mareček R, Fousek J, Strýček O, Rektor I. Large-scale cortico-subcortical functional networks in focal epilepsies: The role of the basal Ganglia. Neuroimage Clin 2016, 14: 28–36. 28123951 10.1016/j.nicl.2016.12.014 PMC5222946

[203] Pang X, Liang X, Zhao J, Wu P, Li X, Wei W, *et al*. Abnormal static and dynamic functional connectivity in left and right temporal lobe epilepsy. Front Neurosci 2021, 15: 820641. 35126048 10.3389/fnins.2021.820641 PMC8813030

[204] Passamonti L, Tsvetanov KA, Jones PS, Bevan-Jones WR, Arnold R, Borchert RJ, *et al*. Neuroinflammation and functional connectivity in Alzheimer’s disease: Interactive influences on cognitive performance. J Neurosci 2019, 39: 7218–7226. 31320450 10.1523/JNEUROSCI.2574-18.2019 PMC6733539

[205] Walker KA, Gross AL, Moghekar AR, Soldan A, Pettigrew C, Hou X, *et al*. Association of peripheral inflammatory markers with connectivity in large-scale functional brain networks of non-demented older adults. Brain Behav Immun 2020, 87: 388–396. 31935468 10.1016/j.bbi.2020.01.006 PMC7316598

[206] Dong X, Hao X, Xu P, Fan M, Wang X, Huang X, *et al*. RNA sequencing analysis of cortex and hippocampus in a kainic acid rat model of temporal lobe epilepsy to identify mechanisms and therapeutic targets related to inflammation, immunity and cognition. Int Immunopharmacol 2020, 87: 106825. 32736192 10.1016/j.intimp.2020.106825

[207] Mirabella F, Desiato G, Mancinelli S, Fossati G, Rasile M, Morini R, *et al*. Prenatal interleukin 6 elevation increases glutamatergic synapse density and disrupts hippocampal connectivity in offspring. Immunity 2021, 54: 2611-2631.e8. 34758338 10.1016/j.immuni.2021.10.006 PMC8585508

[208] Sharma R, Leung WL, Zamani A, O’Brien TJ, Casillas Espinosa PM, Semple BD. Neuroinflammation in post-traumatic epilepsy: Pathophysiology and tractable therapeutic targets. Brain Sci 2019, 9: 318. 31717556 10.3390/brainsci9110318 PMC6895909

[209] Mukherjee S, Arisi GM, Mims K, Hollingsworth G, O’Neil K, Shapiro LA. Neuroinflammatory mechanisms of post-traumatic epilepsy. J Neuroinflammation 2020, 17: 193. 32552898 10.1186/s12974-020-01854-w PMC7301453

[210] Hermann BP, Sager MA, Koscik RL, Young K, Nakamura K. Vascular, inflammatory, and metabolic factors associated with cognition in aging persons with chronic epilepsy. Epilepsia 2017, 58: e152–e156. 28872189 10.1111/epi.13891

[211] Koenig JB, Dulla CG. Dysregulated glucose metabolism as a therapeutic target to reduce post-traumatic epilepsy. Front Cell Neurosci 2018, 12: 350. 30459556 10.3389/fncel.2018.00350 PMC6232824

[212] Liu CH, Yang MH, Zhang GZ, Wang XX, Li B, Li M, *et al*. Neural networks and the anti-inflammatory effect of transcutaneous auricular vagus nerve stimulation in depression. J Neuroinflammation 2020, 17: 54. 32050990 10.1186/s12974-020-01732-5 PMC7017619

[213] Johnson ECB, Dammer EB, Duong DM, Ping L, Zhou M, Yin L, *et al*. Large-scale proteomic analysis of Alzheimer’s disease brain and cerebrospinal fluid reveals early changes in energy metabolism associated with microglia and astrocyte activation. Nat Med 2020, 26: 769–780. 32284590 10.1038/s41591-020-0815-6 PMC7405761

[214] Theodore WH. Presurgical focus localization in epilepsy: PET and SPECT. Semin Nucl Med 2017, 47: 44–53. 27987556 10.1053/j.semnuclmed.2016.09.008

[215] Stredny C, Rotenberg A, Leviton A, Loddenkemper T. Systemic inflammation as a biomarker of seizure propensity and a target for treatment to reduce seizure propensity. Epilepsia Open 2023, 8: 221–234. 36524286 10.1002/epi4.12684 PMC9978091

[216] Farah FH, Grigorovsky V, Bardakjian BL. Coupled oscillators model of hyperexcitable neuroglial networks. Int J Neural Syst 2019, 29: 1850041. 30415633 10.1142/S0129065718500417

[217] Prabowo AS, van Scheppingen J, Iyer AM, Anink JJ, Spliet WG, van Rijen PC, *et al*. Differential expression and clinical significance of three inflammation-related microRNAs in gangliogliomas. J Neuroinflammation 2015, 12: 97. 25986346 10.1186/s12974-015-0315-7 PMC4446114

[218] Strauss KI, Elisevich KV. Brain region and epilepsy-associated differences in inflammatory mediator levels in medically refractory mesial temporal lobe epilepsy. J Neuroinflammation 2016, 13: 270. 27737716 10.1186/s12974-016-0727-z PMC5064886

[219] Zhang Y, Wang Z, Wang R, Xia L, Cai Y, Tong F, *et al*. Conditional knockout of ASK1 in microglia/macrophages attenuates epileptic seizures and long-term neurobehavioural comorbidities by modulating the inflammatory responses of microglia/macrophages. J Neuroinflammation 2022, 19: 202. 35941644 10.1186/s12974-022-02560-5 PMC9361603

[220] Stogsdill JA, Kim K, Binan L, Farhi SL, Levin JZ, Arlotta P. Pyramidal neuron subtype diversity governs microglia states in the neocortex. Nature 2022, 608: 750–756. 35948630 10.1038/s41586-022-05056-7 PMC10502800

[221] Guo X, Kimura A, Namekata K, Harada C, Arai N, Takeda K, *et al*. ASK1 signaling regulates phase-specific glial interactions during neuroinflammation. Proc Natl Acad Sci U S A 2022, 119: e2103812119. 35101972 10.1073/pnas.2103812119 PMC8832969

[222] Vishwakarma S, Singh S, Singh TG. Pharmacological modulation of cytokines correlating neuroinflammatory cascades in epileptogenesis. Mol Biol Rep 2022, 49: 1437–1452. 34751915 10.1007/s11033-021-06896-8

[223] Liu XX, Yang L, Shao LX, He Y, Wu G, Bao YH, *et al*. Endothelial Cdk5 deficit leads to the development of spontaneous epilepsy through CXCL1/CXCR2-mediated reactive astrogliosis. J Exp Med 2020, 217: e20180992. 31699822 10.1084/jem.20180992 PMC7037235

[224] Yamanaka G, Takata F, Kataoka Y, Kanou K, Morichi S, Dohgu S, *et al*. The neuroinflammatory role of pericytes in epilepsy. Biomedicines 2021, 9: 759. 34209145 10.3390/biomedicines9070759 PMC8301485

[225] Ives-Deliperi V, Butler JT. Randomised controlled trial of Naming outcomes in anterior temporal lobectomy versus selective amygdalohippocampectomy. J Neurol Neurosurg Psychiatry 2021, 92: 1020–1021. 33452051 10.1136/jnnp-2020-324531

[226] Jehi L. The epileptogenic zone: Concept and definition. Epilepsy Curr 2018, 18: 12–16. 29844752 10.5698/1535-7597.18.1.12 PMC5963498

[227] Rosenow F, Lüders H. Presurgical evaluation of epilepsy. Brain 2001, 124: 1683–1700. 11522572 10.1093/brain/124.9.1683

[228] Khambhati AN, Davis KA, Lucas TH, Litt B, Bassett DS. Virtual cortical resection reveals push-pull network control preceding seizure evolution. Neuron 2016, 91: 1170–1182. 27568515 10.1016/j.neuron.2016.07.039 PMC5017915

[229] Nicolelis MAL. Brain-machine-brain interfaces as the foundation for the next generation of neuroprostheses. Natl Sci Rev 2022, 9: nwab206. 10.1093/nsr/nwab206 PMC9522427 36196121

[230] Värbu K, Muhammad N, Muhammad Y. Past, present, and future of EEG-based BCI applications. Sensors 2022, 22: 3331. 35591021 10.3390/s22093331 PMC9101004

[231] Piper RJ, Richardson RM, Worrell G, Carmichael DW, Baldeweg T, Litt B, *et al*. Towards network-guided neuromodulation for epilepsy. Brain 2022, 145: 3347–3362. 35771657 10.1093/brain/awac234 PMC9586548

[232] Kringelbach ML, Jenkinson N, Owen SLF, Aziz TZ. Translational principles of deep brain stimulation. Nat Rev Neurosci 2007, 8: 623–635. 17637800 10.1038/nrn2196

[233] Klinger N, Mittal S. Deep brain stimulation for seizure control in drug-resistant epilepsy. Neurosurg Focus 2018, 45: E4. 30064326 10.3171/2018.4.FOCUS1872

[234] Nielsen MS, Bjarkam CR, Sørensen JC, Bojsen-Møller M, Sunde NA, Østergaard K. Chronic subthalamic high-frequency deep brain stimulation in Parkinson’s disease—a histopathological study. Eur J Neurol 2007, 14: 132–138. 17250719 10.1111/j.1468-1331.2006.01569.x

[235] Henderson JM, O’Sullivan DJ, Pell M, Fung VS, Hely MA, Morris JG, *et al*. Lesion of thalamic centromedian—parafascicular complex after chronic deep brain stimulation. Neurology 2001, 56: 1576–1579. 11402120 10.1212/wnl.56.11.1576

[236] Amorim BO, Covolan L, Ferreira E, Brito JG, Nunes DP, de Morais DG, *et al*. Deep brain stimulation induces antiapoptotic and anti-inflammatory effects in epileptic rats. J Neuroinflammation 2015, 12: 162. 26337974 10.1186/s12974-015-0384-7 PMC4558969

[237] Chen Y, Zhu G, Liu D, Zhang X, Liu Y, Yuan T, *et al*. Subthalamic nucleus deep brain stimulation suppresses neuroinflammation by Fractalkine pathway in Parkinson’s disease rat model. Brain Behav Immun 2020, 90: 16–25. 32726685 10.1016/j.bbi.2020.07.035

[238] González HFJ, Yengo-Kahn A, Englot DJ. Vagus nerve stimulation for the treatment of epilepsy. Neurosurg Clin N Am 2019, 30: 219–230. 30898273 10.1016/j.nec.2018.12.005 PMC6432928

[239] Elliott RE, Morsi A, Kalhorn SP, Marcus J, Sellin J, Kang M, *et al*. Vagus nerve stimulation in 436 consecutive patients with treatment-resistant epilepsy: Long-term outcomes and predictors of response. Epilepsy Behav 2011, 20: 57–63. 21144802 10.1016/j.yebeh.2010.10.017

[240] Meneses G, Bautista M, Florentino A, Díaz G, Acero G, Besedovsky H, *et al*. Electric stimulation of the vagus nerve reduced mouse neuroinflammation induced by lipopolysaccharide. J Inflamm 2016, 13: 33. 10.1186/s12950-016-0140-5 PMC5086408 27807399

[241] Namgung U, Kim KJ, Jo BG, Park JM. Vagus nerve stimulation modulates hippocampal inflammation caused by continuous stress in rats. J Neuroinflammation 2022, 19: 33. 35109857 10.1186/s12974-022-02396-z PMC8812005

[242] Picciotto MR, Lewis AS, van Schalkwyk GI, Mineur YS. Mood and anxiety regulation by nicotinic acetylcholine receptors: A potential pathway to modulate aggression and related behavioral states. Neuropharmacology 2015, 96: 235–243. 25582289 10.1016/j.neuropharm.2014.12.028 PMC4486625

[243] Ben-Menachem E, Krauss GL. Epilepsy: Responsive neurostimulation-modulating the epileptic brain. Nat Rev Neurol 2014, 10: 247–248. 24752127 10.1038/nrneurol.2014.69

[244] Kokkinos V, Sisterson ND, Wozny TA, Richardson RM. Association of closed-loop brain stimulation neurophysiological features with seizure control among patients with focal epilepsy. JAMA Neurol 2019, 76: 800–808. 30985902 10.1001/jamaneurol.2019.0658 PMC6583077

[245] Nair DR, Laxer KD, Weber PB, Murro AM, Park YD, Barkley GL, *et al*. Nine-year prospective efficacy and safety of brain-responsive neurostimulation for focal epilepsy. Neurology 2020, 95: e1244–e1256. 32690786 10.1212/WNL.0000000000010154 PMC7538230

[246] Shi J, Lu D, Wei P, Yang Y, Dong H, Jin L, *et al*. Comparative efficacy of neuromodulatory strategies for drug-resistant epilepsy: A systematic review and meta-analysis. World Neurosurg 2024: S1878–S8750(24)01633–4. 10.1016/j.wneu.2024.09.084 39321920

[247] San-Juan D, Espinoza López DA, Vázquez Gregorio R, Trenado C, Fernández-González Aragón M, Morales-Quezada L, *et al*. Transcranial direct current stimulation in mesial temporal lobe epilepsy and hippocampal sclerosis. Brain Stimul 2017, 10: 28–35. 27693237 10.1016/j.brs.2016.08.013

[248] Rabenstein M, Unverricht-Yeboah M, Keuters MH, Pikhovych A, Hucklenbroich J, Vay SU, *et al*. Transcranial current stimulation alters the expression of immune-mediating genes. Front Cell Neurosci 2019, 13: 461. 31708742 10.3389/fncel.2019.00461 PMC6824260

[249] Guo B, Zhang M, Hao W, Wang Y, Zhang T, Liu C. Neuroinflammation mechanisms of neuromodulation therapies for anxiety and depression. Transl Psychiatry 2023, 13: 5. 36624089 10.1038/s41398-022-02297-y PMC9829236

[250] Yang JC, Bullinger KL, Dickey AS, Karakis I, Alwaki A, Cabaniss BT, *et al*. Anterior nucleus of the thalamus deep brain stimulation vs temporal lobe responsive neurostimulation for temporal lobe epilepsy. Epilepsia 2022, 63: 2290–2300. 35704344 10.1111/epi.17331 PMC9675907

[251] Ryvlin P, Rheims S, Hirsch LJ, Sokolov A, Jehi L. Neuromodulation in epilepsy: State-of-the-art approved therapies. Lancet Neurol 2021, 20: 1038–1047. 34710360 10.1016/S1474-4422(21)00300-8

[252] Scangos KW, Khambhati AN, Daly PM, Makhoul GS, Sugrue LP, Zamanian H, *et al*. Closed-loop neuromodulation in an individual with treatment-resistant depression. Nat Med 2021, 27: 1696–1700. 34608328 10.1038/s41591-021-01480-w PMC11219029

[253] Patel UK, Anwar A, Saleem S, Malik P, Rasul B, Patel K, *et al*. Artificial intelligence as an emerging technology in the current care of neurological disorders. J Neurol 2021, 268: 1623–1642. 31451912 10.1007/s00415-019-09518-3

[254] Beniczky S, Karoly P, Nurse E, Ryvlin P, Cook M. Machine learning and wearable devices of the future. Epilepsia 2021, 62: S116–S124. 32712958 10.1111/epi.16555

[255] Nosi D, Lana D, Giovannini MG, Delfino G, Zecchi-Orlandini S. Neuroinflammation: Integrated nervous tissue response through intercellular interactions at the “whole system” scale. Cells 2021, 10: 1195. 34068375 10.3390/cells10051195 PMC8153304

[256] Ureña-Guerrero ME, Castañeda-Cabral JL, Rivera-Cervantes MC, Macias-Velez RJ, Jarero-Basulto JJ, Gudiño-Cabrera G, *et al*. Neuroprotective and neurorestorative effects of epo and VEGF: Perspectives for new therapeutic approaches to neurological diseases. Curr Pharm Des 2020, 26: 1263–1276. 31942853 10.2174/1381612826666200114104342

[257] Marchi N, Lerner-Natoli M. Cerebrovascular remodeling and epilepsy. Neuroscientist 2013, 19: 304–312. 23072899 10.1177/1073858412462747 PMC3701121

[258] Beamer E, Fischer W, Engel T. The ATP-gated P2X7 receptor As a target for the treatment of drug-resistant epilepsy. Front Neurosci 2017, 11: 21. 28210205 10.3389/fnins.2017.00021 PMC5288361

[259] Xu X, Zhang A, Zhu Y, He W, Di W, Fang Y, *et al*. MFG-E8 reverses microglial-induced neurotoxic astrocyte (A1) via NF-κB and PI3K-Akt pathways. J Cell Physiol 2018, 234: 904–914. 30076715 10.1002/jcp.26918

[260] Stephan AH, Barres BA, Stevens B. The complement system: An unexpected role in synaptic pruning during development and disease. Annu Rev Neurosci 2012, 35: 369–389. 22715882 10.1146/annurev-neuro-061010-113810

[261] Lian H, Litvinchuk A, Chiang ACA, Aithmitti N, Jankowsky JL, Zheng H. Astrocyte-microglia cross talk through complement activation modulates amyloid pathology in mouse models of Alzheimer’s disease. J Neurosci 2016, 36: 577–589. 26758846 10.1523/JNEUROSCI.2117-15.2016 PMC4710776

[262] Soares LC, Al-Dalahmah O, Hillis J, Young CC, Asbed I, Sakaguchi M, *et al*. Novel galectin-3 roles in neurogenesis, inflammation and neurological diseases. Cells 2021, 10: 3047. 34831271 10.3390/cells10113047 PMC8618878

[263] Rotshenker S, Reichert F, Gitik M, Haklai R, Elad-Sfadia G, Kloog Y. Galectin-3/MAC-2, Ras and PI3K activate complement receptor-3 and scavenger receptor-AI/II mediated myelin phagocytosis in microglia. Glia 2008, 56: 1607–1613. 18615637 10.1002/glia.20713

[264] Jansen MI, Thomas Broome S, Castorina A. Exploring the pro-phagocytic and anti-inflammatory functions of PACAP and VIP in microglia: Implications for multiple sclerosis. Int J Mol Sci 2022, 23: 4788. 35563181 10.3390/ijms23094788 PMC9104531

[265] Hu Y, Yao Y, Qi H, Yang J, Zhang C, Zhang A, *et al*. Microglia sense and suppress epileptic neuronal hyperexcitability. Pharmacol Res 2023, 195: 106881. 37541638 10.1016/j.phrs.2023.106881

[266] Pawelec P, Ziemka-Nalecz M, Sypecka J, Zalewska T. The impact of the CX3CL1/CX3CR1 axis in neurological disorders. Cells 2020, 9: 2277. 33065974 10.3390/cells9102277 PMC7600611

[267] Roseti C, Fucile S, Lauro C, Martinello K, Bertollini C, Esposito V, *et al*. Fractalkine/CX3CL1 modulates GABAA currents in human temporal lobe epilepsy. Epilepsia 2013, 54: 1834–1844. 24032743 10.1111/epi.12354

[268] Zaitsev AV, Smolensky IV, Jorratt P, Ovsepian SV. Neurobiology, functions, and relevance of excitatory amino acid transporters (EAATs) to treatment of refractory epilepsy. CNS Drugs 2020, 34: 1089–1103. 32926322 10.1007/s40263-020-00764-y

[269] Lee SG, Su ZZ, Emdad L, Gupta P, Sarkar D, Borjabad A, *et al*. Mechanism of ceftriaxone induction of excitatory amino acid transporter-2 expression and glutamate uptake in primary human astrocytes. J Biol Chem 2008, 283: 13116–13123. 18326497 10.1074/jbc.M707697200 PMC2442320

[270] Chen P, Chen F, Zhou B. Understanding the role of *Glia*-neuron communication in the pathophysiology of epilepsy: A review. J Integr Neurosci 2022, 21: 102. 35864754 10.31083/j.jin2104102

[271] Muraleedharan R, Gawali MV, Tiwari D, Sukumaran A, Oatman N, Anderson J, *et al*. AMPK-regulated astrocytic lactate shuttle plays a non-cell-autonomous role in neuronal survival. Cell Rep 2020, 32: 108092. 32877674 10.1016/j.celrep.2020.108092 PMC7531170

[272] Barros LF. Metabolic signaling by lactate in the brain. Trends Neurosci 2013, 36: 396–404. 23639382 10.1016/j.tins.2013.04.002

[273] Rogawski MA. Astrocytes get in the act in epilepsy. Nat Med 2005, 11: 919–920. 16145568 10.1038/nm0905-919 PMC1373800

